# Temporal Variation in Bacterial Community Characteristics Shaped by Habitat in a Reservoir Buffer Strip in China

**DOI:** 10.1002/ece3.70957

**Published:** 2025-03-07

**Authors:** Tengfei Yan, Zhengxin Wang, Zhen Wang, Yong Qin, Zheng Wang, Songwei Li

**Affiliations:** ^1^ Xinyang Huai River Catchment Riparian Zone Carbon Neutralization Engineering Technology Xinyang Agriculture and Forestry University Xinyang China; ^2^ Xinyang Ecological Research Institute Xinyang China; ^3^ Henan Province Engineering Research Center of Biological Pesticide & Fertilizer Development and Synergistic Application Henan Institute of Science and Technology Xinxiang China; ^4^ School of Landscape Architecture and Art Henan Agriculture University Zhengzhou China

**Keywords:** bipartite network, community stability, co‐occurrence network, reservoir buffer zone, soil properties, spatiotemporal variation

## Abstract

Habitat and temporal variation can both influence microbial community dynamics, although their relative importance in reservoir buffer zones with complex hydrology regimes and dramatically altered environments remains controversial. To elucidate this, we investigated spatiotemporal variation in soil bacterial diversity and ecological processes from the flooding period to the dry period (April and June, respectively) using high‐throughput 16S amplicon sequencing in three habitats (abandoned cropland, grassland, and woodland) within the Chushandian Reservoir's buffer strip, China. The results showed that habitat was more important than temporal variation in shaping soil bacterial diversity and ecological processes in the reservoir buffer zone. Bacterial communities responded to temporal variation both in terms of species composition and function; temporal variation affected bacterial communities mostly by altering the abundance of shared species and by causing the resurgence or extinction of specific taxa within the same habitat. The main driver of these changes was the resilience capacity of habitats to the changing moisture environment. The magnitude and underlying mechanisms of the changes in bacterial community diversity and ecological processes differed markedly between the three habitats, owing mostly to the characteristics of their vegetation, thus the allocation ratios of different habitat vegetation types and landscape diversity should pay attention for the reservoir buffer zone management, improving the integrated ecological benefits of the reservoir ecosystem from a multiscale.

## Abstract 2

Habitat is more important than temporal variation in shaping soil bacterial community diversity in reservoir buffer strips; the adaptive capacity of habitats to changes in the moisture environment is the main driver. Changes in the abundance of shared species and resurgence or extinction of some specific taxa in the same habitat.

## 1 Introduction

The reservoir buffer zone bridges aquatic and terrestrial ecosystems, playing an essential ecological role in maintaining water quality, blocking upland agriculture non‐point source pollution, and enhancing landscape diversity, all of which are closely related to reservoir management and conservation strategies (Saleh et al. 2018; Cole et al. 2020). As one of the most prevalent and widespread microbial communities in the reservoir buffer zone, soil bacteria not only participate in a variety of biogeochemical cycles, but also co‐evolve with their habitats to promote ecosystem health and stability (Evans and Wallenstein 2014; Wu et al. 2022; Philippot et al. 2023). Multiple studies have shown that, while shifts in bacterial community composition occur in response to environmental changes, they also serve as predictors of ecosystem functions and processes (De Vries and Shade 2013; Yang et al. 2019; Yuan et al. 2021). Therefore, studying the synergistic relationships between bacterial community composition and environmental changes is of great relevance for understanding microbial community dynamics and function.

Clarifying the relative importance of spatial and temporal variation in shaping soil bacterial communities can provide deeper insight into the synergistic relationships between microbial community dynamics and the environment (Ladau and Eloe‐Fadrosh 2019). The reservoir buffer zone is considered an ideal place to study soil bacterial communities over time and space because it provides a continuous moisture gradient with a wide range of habitats (Fournier et al. 2020; Saleh et al. 2018; Cole et al. 2020). Alternating dry‐wet soil moisture conditions owing to water level fluctuations are the main drivers of reservoir bank habitat change (Annala et al. 2022). Changes in moisture content directly reflect the availability of oxygen in the reservoir bank soil environment, causing bacterial communities to switch between aerobic and anaerobic conditions, thus affecting soil chemical processes and altering physicochemical parameters (Harrison 2008; De Nijs et al. 2019; Zhang, Lin et al. 2021).

The complex moisture environment of the reservoir buffer zone affects bacterial communities at both the temporal and spatial scales. Interseasonally driven changes in soil bacterial communities in reservoir buffer zones are thought to be caused by changes in environmental parameters (Wang et al. 2019; De Gruyter et al. 2020). Interannual water‐level fluctuations gradually simplify the soil bacterial community, primarily via environmental filtering by the ecological conditions of the reservoir buffer zone (Yang et al. 2019; Zheng et al. 2021; Jiajia et al. 2023). Spatially, the hydraulic gradient caused by the underground water level profoundly affects the distribution of flora and fauna in the reservoir buffer zone (Dybkjær et al. 2012; Annala et al. 2022). For example, with increasing distance from the watercourse, the soil moisture content in the reservoir buffer zone gradually decreases and bacterial and fungal biomass (PLFA) gradually increases (Wang et al. 2019).

Habitat is another important factor influencing the spatial distribution of soil bacterial communities in the reservoir buffer zone (Rampelotto et al. 2013; Ding et al. 2022). The quantity and quality of litter and the recruitments of plant roots for specific microorganisms reflect the influence of aboveground vegetation on soil bacterial community composition (Yan et al. 2018; Jiajia et al. 2023). Zhang, O'Connor et al. (2021) suggested that vegetation coverage shapes reservoir buffer zone bacterial communities by improving nutrient availability and retention. In contrast, for residual woodland, reforestation, and pasture in reservoir buffer zones, Waymouth et al. (2021) found that soil bacterial community composition and function were more directly influenced by soil properties than by differences in vegetation. These contradictory results imply that there may be complex spatiotemporal relationships between vegetation and soil bacterial communities in the reservoir buffer zone, requiring further investigation (Regan et al. 2014).

There is a shortage of data on both the temporal and spatial variability of soil bacterial communities in the reservoir buffer zone. Further, the reservoir buffer zone has complex hydrological characteristics, as they include artificial and natural zones and experience dry and wet periods, for instance. Consequently, research on these dynamics should be exceptionally cautious, because the impacts of temporal variation and fluctuating water levels are intertwined, and this can easily generate results that are difficult to interpret (Hermans et al. 2020). For a restored floodplain, Samaritani et al. (2017) suggested that soil bacterial communities changed more substantially between seasons than between habitats. Notably, their study examined habitats at a range of distances from the watercourse; based on spatial heterogeneity owing to the moisture gradient, the effects of temporal variation on these bacterial communities may have been overestimated. In other words, the resilience capacity (the capacity of a system to resist and recover from disturbance; Riva et al. 2023) of bacterial communities in different habitats to environmental disturbances was attributed to the effect of temporal variation.

Reservoir buffer strip, as an important component of the reservoir buffer zone, is the unit of implementation of physical management measures that is directly connected to the watershed and is also referred to as the vegetated buffer strip, which is often confused with the reservoir buffer zone in much of the literature (Stutter et al. 2019; Cole et al. 2020). For the purposes of this study, we specifically refer to the vegetated buffer strip of different habitats, which is the last barrier to maintain reservoir habitat and is the key area for reservoir restoration and management. The width setting of the reservoir buffer strip is controversial depending on the ecological purpose, yet it is generally consensus that it should be maintained at 10–30 m and can be considered to be in the same hydraulic gradient as the macroscopic reservoir buffer zone.

We hypothesized that the spatiotemporal variability of soil bacterial communities in reservoir buffer zones is primarily ascribed to the resilience capacity of different habitats to environmental disturbances and further results in differences in the abundance, functionality, and ecology niche width of the bacterial taxa. To test this hypothesis, reservoir buffer strips containing different vegetation types were selected as the research object. The soil bacterial community species composition, distribution characteristics, and ecological processes in the flooding and dry periods (April and June, respectively) were focused on in order to elucidate the mechanisms by which habitat and temporal variation affect soil bacterial community dynamics in the reservoir buffer zone. The main aims of this study were: (1) to clarify the importance of relative contributions of habitat and temporal variation in shaping soil bacterial community changes in reservoir buffer zones. (2) to reveal how relatively rapid environmental changes in the reservoir buffer strip alter the composition, abundance, diversity, and ecological function potential of bacterial communities. (3) to discuss the potential regulatory mechanism behind these changes and the practical significance for reservoir bank management.

## 2 Materials and Methods

### 2.1 Site Description and Soil Sampling

**FIGURE 1:**
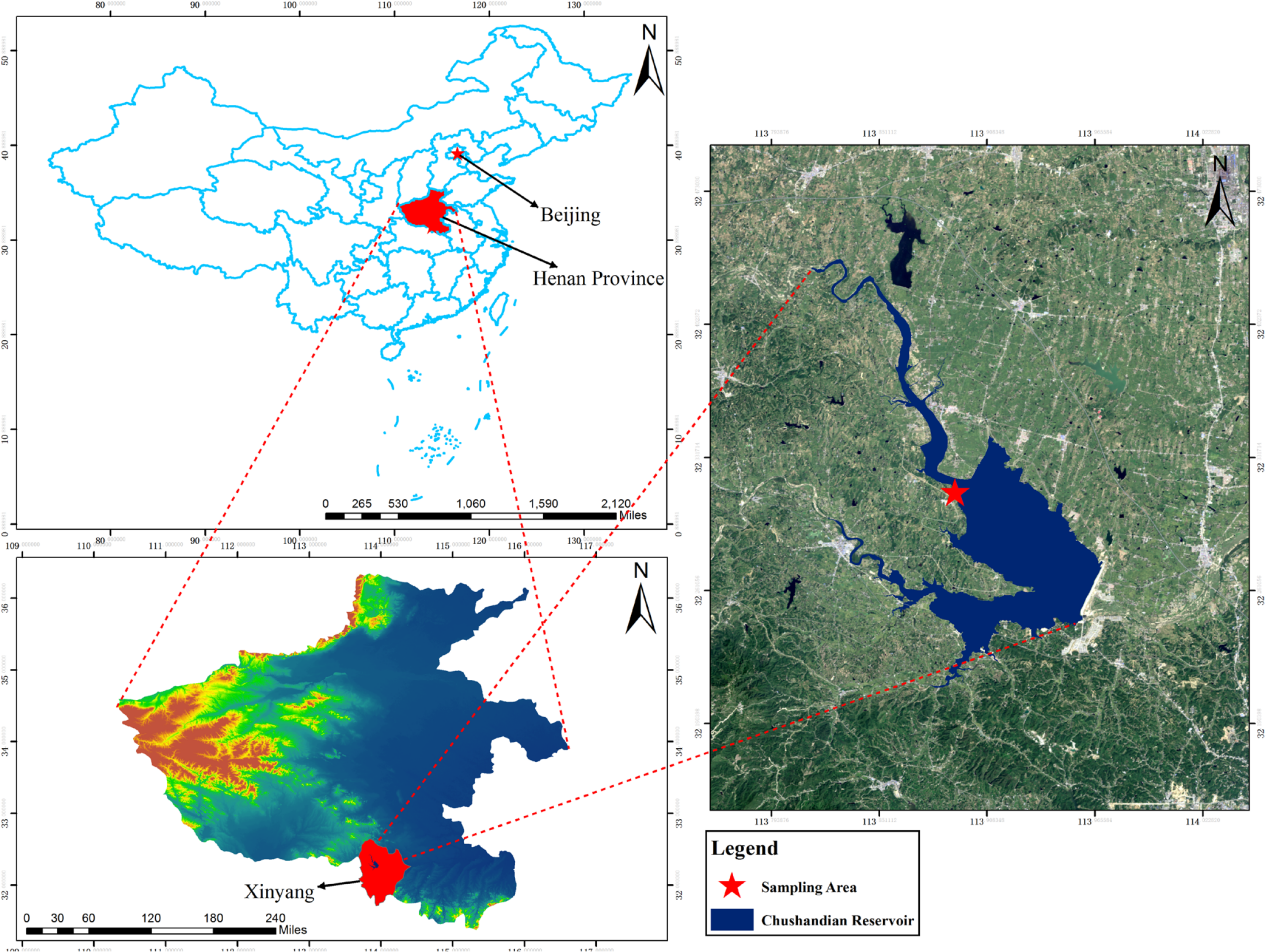
The sampling area is located in the Chushandian Reservoir's buffer strips (C), Henan province (B), and China (A).

The study was conducted at the Chushandian Reservoir (N 32°22′‐32°32′, E 113°89′‐113°96′), Henan province, China, in 2021 (Figure 1). The Chushandian Reservoir is a newly built regulated storage reservoir that was first impounded in May 2019. The climatic conditions and reservoir operating status are described in our previous article (Yan et al. 2023). The Chushandian reservoir is a typical subtropical reservoir of “winter storage and summer discharge” with an obvious dry period (from May to September) and a flooding period (from October to the following April). As a large amount of cultivated land was occupied during the construction of the reservoir, by the time the reservoir was in operation, the reservoir's water bank had formed a patchy landscape dominated by woodland, grassland, and abandoned cropland, and the length of individual landscapes is 20–30 m away from the watercourse.

During the flooding period, three habitats (woodlands, grasslands, and abandoned croplands) immediately adjacent to the watercourse were selected as fixed sample plots along the normal water level line to ensure that each habitat was on the same hydraulic gradient, with a distance of no more than 1 km between habitats. The woodland vegetation is dominated by *Populus*, with occasional *Chionanthus retusus*. The grassland comprised mainly *Imperata cylindrica* . The abandoned cropland was cultivated before the construction of the reservoir but was abandoned for two years at the time of sampling. The vegetation is mainly herbaceous, and its composition changes rapidly with fluctuations in the water level.

**FIGURE 2:**
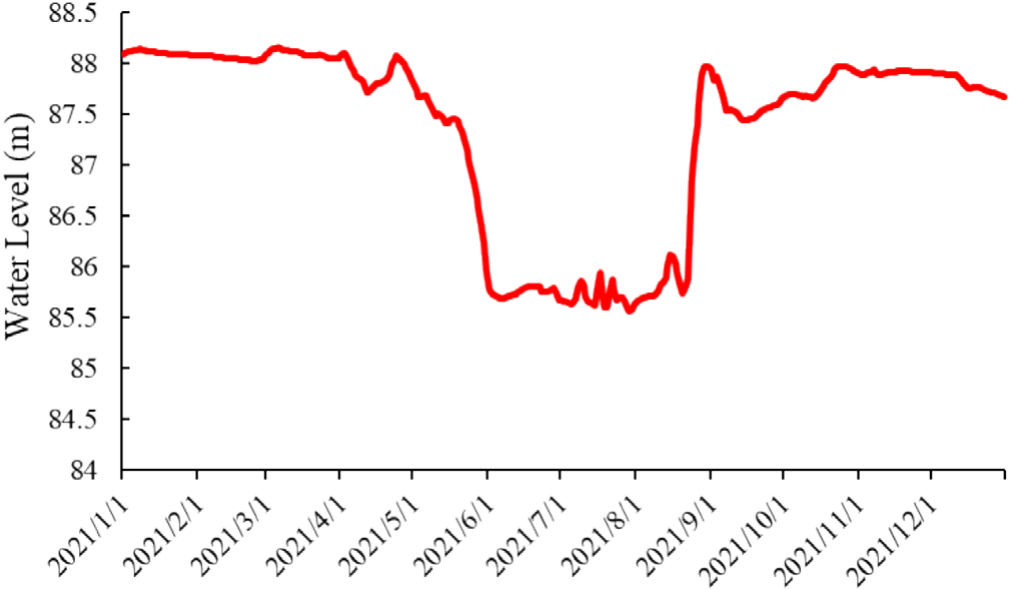
The water level fluctuation rhythm of Chushandian Reservoir in 2021.

Soil sampling was conducted on April 13 and June 8, approximately one month before and one month after, respectively, the reservoir's first discharge of the year (May 12), to ensure that soil microbes reached a steady state (Figure 2) and avoid the possible effects of rainy season precipitation after June. In each habitat, four sample strips were set up perpendicular to the water‐table line, at a distance of not less than 50 m. Three sampling points were set up along the sample strips (at 2, 10, and 20 m from the watercourse, due to the fragmentation of water‐bank habitats; independent habitats have a maximum of 30 m). Due to this study focused on habitat, we ignored the possible effects of hydraulic gradients at fine distances in the reservoir buffer strip and characterized habitat as a whole by taking soil sampling points collected at three different distances. 12 samples were collected from each habitat, with a total of 72 soil samples collected over two periods (three habitats × four sample strips × three sampling points × two periods).

Five soil samples were randomly collected at each sampling point and mixed into one homogeneous sample, with a sampling depth of 0–20 cm. Each homogenized sample was divided into two; one half was placed in a self‐sealing bag for the determination of physical and chemical properties, and the other half was placed in a sterile sampling tube on ice, brought back to the laboratory, and stored in a refrigerator at −80°C for identification of soil microorganisms.

### 2.2 Soil Physicochemical Analysis

Soil pH was determined using the electrode potential method using a pH meter (ST3100/F, U.S.). The water‐to‐soil ratio was 2.5:1. Soil total carbon (TC, g/kg) and total nitrogen (TN, g/kg) were determined using a Vario Max CNS elemental analyzer (Elementar Analysensysteme GmbH, Hanau, Germany). Total phosphorus (TP, g/kg) was determined using the Mo‐Sb colorimetric method. Ammonium‐nitrogen (NH_4_‐N, mg/kg) and nitrate‐nitrogen (NO_3_‐N, mg/kg) content was determined using an Automatic Discontinuous Chemical Analyzer (Smarthem200, Alliance Company, France). Dissolved organic carbon (DOC, mg/kg) content was determined using a Total Organic Carbon (TOC) analyzer (Multi C/N 2100, Analytik Jena, Jena, Germany) after extraction using K_2_SO_4_ and filtering through a 0.45 μm membrane. Microbial biomass carbon (MBC, mg/kg) and microbial biomass nitrogen (MBN, mg/kg) were determined using the fumigation‐extraction method. Soil bulk density (BD, g/cm^3^) was determined using the ring‐cutting method. Soil water content (SWC, %) was determined by drying the soil to a constant weight at 80°C.

### 2.3 DNA Extraction, PCR Amplification, and Sequencing

Microbial DNA was extracted from soil samples using the E.Z.N.A. soil DNA Kit (Omega Bio‐tek, Norcross, GA, U.S.) according to the manufacturer's protocols. The final DNA concentration and purity were determined using a NanoDrop 2000 UV–vis spectrophotometer (Thermo Fisher Scientific, Waltham, MA, USA), and DNA quality was checked via 1% agarose gel electrophoresis. The V3‐V4 hypervariable regions of the bacterial 16S rRNA gene were amplified via PCR using primers 338F/806R on a GeneAmp 9700 thermocycler (ABI; Thermo Fisher Scientific). The resulting PCR products were extracted from a 2% agarose gel and further purified using the AxyPrep DNA Gel Extraction Kit (Axygen Biosciences, Union City, CA, USA) and quantified using a QuantiFluor‐ST fluorometer (Promega, Madison, WI, USA), according to the manufacturer's protocol. Purified amplicons were pooled in equimolar concentrations and paired‐end sequenced (2 × 300 bp) on an Illumina MiSeq platform (Illumina, San Diego, CA, USA), according to the standard protocols of Majorbio Bio‐Pharm Technology Co. Ltd. (Shanghai, China). The data were deposited at the National Microbiology Data Center (NMDC) under accession number NMDC10018801.

### 2.4 Bioinformatic Analysis

A total of 4,906,538 sequences were obtained for the 72 samples. The raw sequences were processed using the QIIME2 pipeline, quality control, chimera removal, and denoised using DADA2. Amplicon Sequence Variants (ASVs) were obtained based on a 99% similarity threshold. Finally, a total of 1,396,547 ASVs were kept, and each sample had about 14,100–24,132 ASVs (with a median depth of 19,630). Taxonomic classification was performed using the SILVA database (http://www.arb‐silva.de/) as a reference. We used the metagenome prediction tool PICRUST2 to predict the functional profiles of the microbial communities based on their taxonomic composition. The ASV data were normalized by count, and the predicted functional gene community was generated using the Kyoto Encyclopedia of Genes and Genomes (KEGG) database. The predicted functional metagenomes were then classified at KEGG level 3.

### 2.5 Statistical and Data Analyses

All statistical analyses were performed using RStudio (version 4.2). The effects of habitat, temporal variation, and their interaction on soil physicochemical properties and microbial *α*‐diversity indices were tested by two‐way ANOVA followed by Tukey test, using the R package *multcompView* labeled significant difference. Principal Component Analysis (PCA) was used to examine the spatiotemporal patterns of soil's physicochemical properties between different periods by using the R package *FactoMineR*. Variation partition analysis (VPA) was used to determine the effects of habitat, temporal variation, soil microbial properties, and soil physicochemical properties on microbial community *β*‐diversity by using the R package *rdacca.hp* (Lai et al. 2022). Soil microbial properties included MBC, MBN, and DOC. Soil physicochemical properties included BD, SWC, TC, TN, TP, NH_4_‐N, and NO_3_‐N. The VPA results were visualized using the R package *UpSetR*.

To account for differences in the number of reads, the bacterial datasets were rarefied to 14,000 reads per sample using the rrarefy function of the *Vegan* package. Principal coordinate analysis (PCoA) was used to examine the spatiotemporal patterns of the bacterial communities for different habitats and periods in the reservoir buffer strip, based on Bray‐Curtis and weighted‐UniFrac distances, respectively, by using the cmdscale function of the *Vegan* package. Analysis of similarity (ANOSIM) was used to verify the strength and statistical significance of the similarities in microbial communities among the habitats and periods and to evaluate the combined effects of habitat and period. *β*‐diversity was analyzed using permutational multivariate analysis of variance (PERMANOVA) with 999 random permutations (*p* < 0.05), using the adonis function of the *Vegan* package. Constrained principle coordinated analysis (CPA) was performed using the function capscale of the *Vegan* package to explore the effects of environmental factors on the bacterial communities for different habitats and periods in the reservoir buffer strip; forward selection was first applied by using the function ordiR2step to lessen the influence of factor collinearity. The wilcox.test function in R was used to identify significant family‐level changes in bacterial community composition between the two periods within each habitat and visualized using the R package *pheatmap*; this function was also used to determine the significance of the difference in predicted ecological function between the periods within each habitat and visualized using the R package *ggplot2*. The Mantel test was used to test the relationship between the top 10 abundant bacterial taxa (phylum level) and soil physicochemical properties at the two periods, respectively, visualized using the R package *LinkET*.

Complementary approaches were employed to identify the ASVs with significant response between different habitats and periods (Hartman et al. 2018). To reduce the impact of site‐specific ASVs and to facilitate in‐depth assessment of each bacterial community, we selected ASVs with at least two sequences (avoiding single‐count ASVs) present in at least four samples. We applied correlation‐based indicator species analysis by using the R package *indicspecies*, conducted using 10^4^ permutations; correlations were considered significant at *p* < 0.05. The likelihood ratio test (LRT) was applied by using the R package *edgeR*, requiring a corrected false discovery rate of *p* < 0.05; we then defined ASVs that were confirmed by both indicator species analysis and LRT as exhibiting significant variation taxa between habitats or with the habitat‐period combination. A bipartite network was used to visualize significant associations (*p* < 0.05) of these ASVs with habitat or with a combination of habitat and period, based on the indicator species analysis. The bipartite network was constructed using Gephi.

Also using Gephi, Spearman rank correlations between pairs of bacterial ASVs were calculated, with thresholds of *r* > 0.6 and *p* < 0.05, to construct the co‐occurrence network for each habitat at each period. Descriptive and topological network properties were calculated using the R package *igraph*. Within‐module connectivity (*Zi*) and among‐module connectivity (*Pi*) were calculated to evaluate node‐connection strength within and between modules, respectively. The ASVs were divided into four categories: network hubs (*Zi* > 2.5, *Pi* > 0.62), module hubs (*Zi* > 2.5, *Pi* < 0.62), connectors (*Zi* < 2.5, *Pi* > 0.62), and peripherals (*Zi* < 2.5, *Pi* < 0.62) (Olesen et al. 2006).

The modified stochasticity ratio (MST), which was a special form of normalized stochasticity ratio (NST) assumption that observed similarity can be equal to the mean of null similarity under pure stochastic assembly, was used to determine the potential importance of stochastic and deterministic processes in bacterial community assembly for habitats in the different periods, using the R package *NST* (Ning et al. 2019; An et al. 2023). MST > 0.5 indicates that the processes are primarily stochastic, while MST < 0.5 indicates that they are primarily deterministic; average niche width was calculated to assess the adaptability of the bacterial community to the environment in different habitats, using the R package *spaa*. The size of niche breadth represents the metabolic flexibility of species, and a wider niche breadth indicates that a species has better environmental adaptability and is more likely to survive stably in a larger time or space (Xiong et al. 2020); Average Variation Degree (AVD) was used to assess the bacterial community stability of habitats in the different periods. AVD, which exponentiates community stability, has the advantage of not being limited by the number of samples within a group (Xun et al. 2021). The smaller the AVD value, the more stable the microbial community is.

Partial least squares structure equation modeling (PLS‐SEM) was conducted to explore the potential mechanisms through which habitats modulate bacterial community resilience in response to temporal variation, using the R package *plspm*. The hypothesized model structure was based on habitat and temporal variation, which can both determine bacterial community resilience directly and by influencing soil properties and bacterial community diversity. Habitat and temporal variation were defined as nominal variables. Soil properties, bacterial community diversity, and bacterial community resilience were constructed as latent variables. Soil physicochemical factors extracted from CAP analysis results were used to construct soil properties; Shannon diversity and principal component characteristics of the first axis of PCoA analysis results were used to construct bacterial community diversity; niche width and AVD were used to construct bacterial community resilience.

## 3 Results

### 3.1 Spatiotemporal Variation in Soil Physicochemical Properties

Between the flooding period (April) and the dry period (June), soil pH changed significantly for woodland (*p* < 0.001), from alkaline (7.63) to acidic (6.13), and for grassland (*p* < 0.001), from acidic (6.05) to alkaline (7.74). TN increased significantly for the grassland (*p* = 0.0025), but not in the other two habitat types. TC decreased significantly for abandoned cropland (*p* = 0.026) and woodland (*p* < 0.001). TP decreased for grassland (*p* < 0.001) and increased significantly for woodland (*p* < 0.001). NH_4_‐N and NO_3_‐N increased significantly in all three habitats, with the exception of NO_3_‐N in woodland (*p* = 0.99). DOC and MBC increased significantly in woodland (*p* < 0.001; *p* < 0.001). MBN increased significantly in woodland (*p* = 0.001) and grassland (*p* = 0.006). BD decreased in woodland (*p* = 0.102) and abandoned cropland (*p* = 0.003), increased in grassland (*p* = 0.132). SWC decreased significantly in all three habitats (Table 1). PCA revealed clear temporal differentiation in the soil physicochemical properties of the three habitats in the reservoir buffer strips: within each habitat, the two periods were each distributed on both sides of the axes, and the dispersion between the habitats was significantly greater in the dry period than in the flooding period (Figure S1).

**TABLE 1:** Soil physicochemical properties of the reservoir‐buffer‐strip habitats in the flooding period (April) and dry period (June). Different lowercase letters represent the significant difference among different habitats.

Month	Cropland		Grassland		Woodland	
	April	June	April	June	April	June
pH	5.98 ± 0.69^b^	6.43 ± 0.6^b^	6.05 ± 0.25^b^	7.74 ± 0.4^a^	7.63 ± 0.57^a^	6.13 ± 0.19^b^
TN (g/kg)	0.48 ± 0.13^b^	0.65 ± 0.13^ab^	0.48 ± 0.13^b^	0.73 ± 0.16^a^	0.54 ± 0.16^ab^	0.64 ± 0.2^ab^
TC (g/kg)	8.1 ± 1.58^b^	6.62 ± 1.45^c^	8.78 ± 1.95^ab^	8.78 ± 1.51^ab^	10.41 ± 1.9^a^	6.83 ± 1.03^bc^
TP (g/kg)	0.38 ± 0.06^bc^	0.41 ± 0.07^bc^	0.32 ± 0.02^cd^	0.48 ± 0.04^a^	0.45 ± 0.05^ab^	0.34 ± 0.03^c^
NH_4_‐N (mg/kg)	13.79 ± 2.16^c^	32.05 ± 10.26^a^	7.12 ± 1.35^d^	19.4 ± 4.78^bc^	11.58 ± 1.81^d^	23.92 ± 5.94^b^
NO_3_‐N (mg/kg)	0.95 ± 0.43^c^	3.29 ± 1.38^b^	0.72 ± 0.12^c^	6.62 ± 1.78^a^	4.62 ± 1.39^b^	4.31 ± 1.62^b^
DOC (mg/kg)	13.15 ± 2.3^c^	13.84 ± 2.58^bc^	17 ± 2.82^b^	17.15 ± 3.32^b^	16.92 ± 2.45^b^	22.97 ± 4.66^a^
MBC (mg/kg)	112.36 ± 24.55^d^	139.38 ± 22.23^cd^	228.02 ± 42.07^ab^	185.92 ± 42.11^bc^	171.32 ± 43.37^bcd^	279.58 ± 105.56^a^
MBN (mg/kg)	12.03 ± 2.44^d^	12.82 ± 3.04^d^	26.17 ± 6.93^bc^	34.49 ± 7.1^a^	22.09 ± 5.71^c^	31.68 ± 6.24^ab^
BD (g/cm^3^)	1.55 ± 0.24^ab^	1.23 ± 0.22^c^	1.4 ± 0.2^abc^	1.62 ± 0.22^a^	1.54 ± 0.21^ab^	1.31 ± 0.16^bc^
SWC (%)	19.82 ± 3.18^ab^	9.77 ± 1.27^c^	20.39 ± 2.01^a^	16.97 ± 3.22^b^	18.12 ± 1.61^ab^	10.9 ± 2.63^c^

### 3.2 Spatiotemporal Variation in Soil Bacterial Community Diversity

**FIGURE 3:**
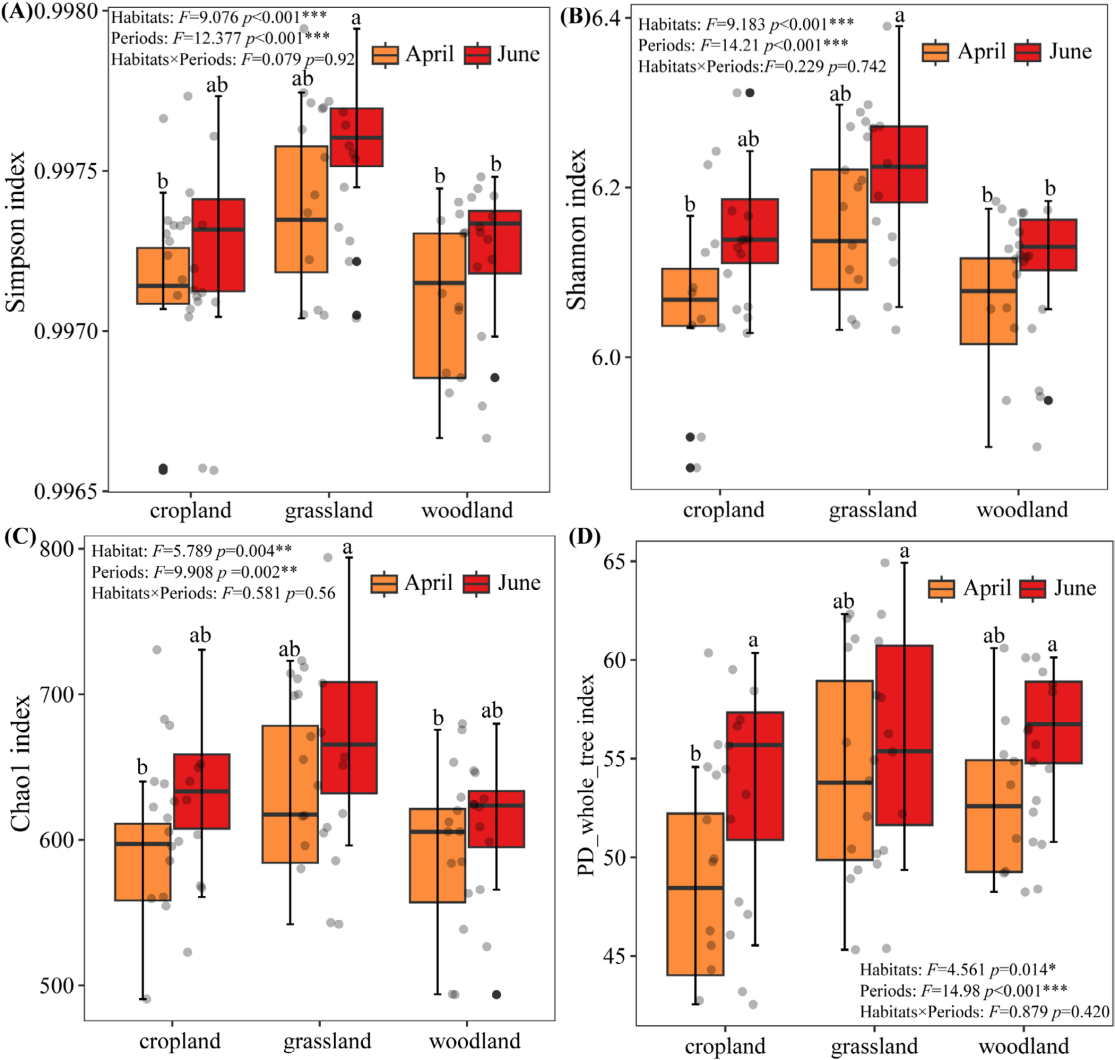
Bacterial community biodiversity of the different reservoir‐buffer‐strip habitats in the flooding period (April) and dry period (June). (A) *Simpson* index (B) *Shannon* index (C) *Chao1* index (D) *PD‐whole‐tree* index.

Soil bacterial community *α*‐diversity indices were greater in the dry period than in the flooding period. Within each period, *α*‐diversity indices were highest for grassland (although not significantly). Two‐way *ANOVA* revealed that all bacterial community diversity indices were significantly affected by habitat and period (Figure 3).

**FIGURE 4:**
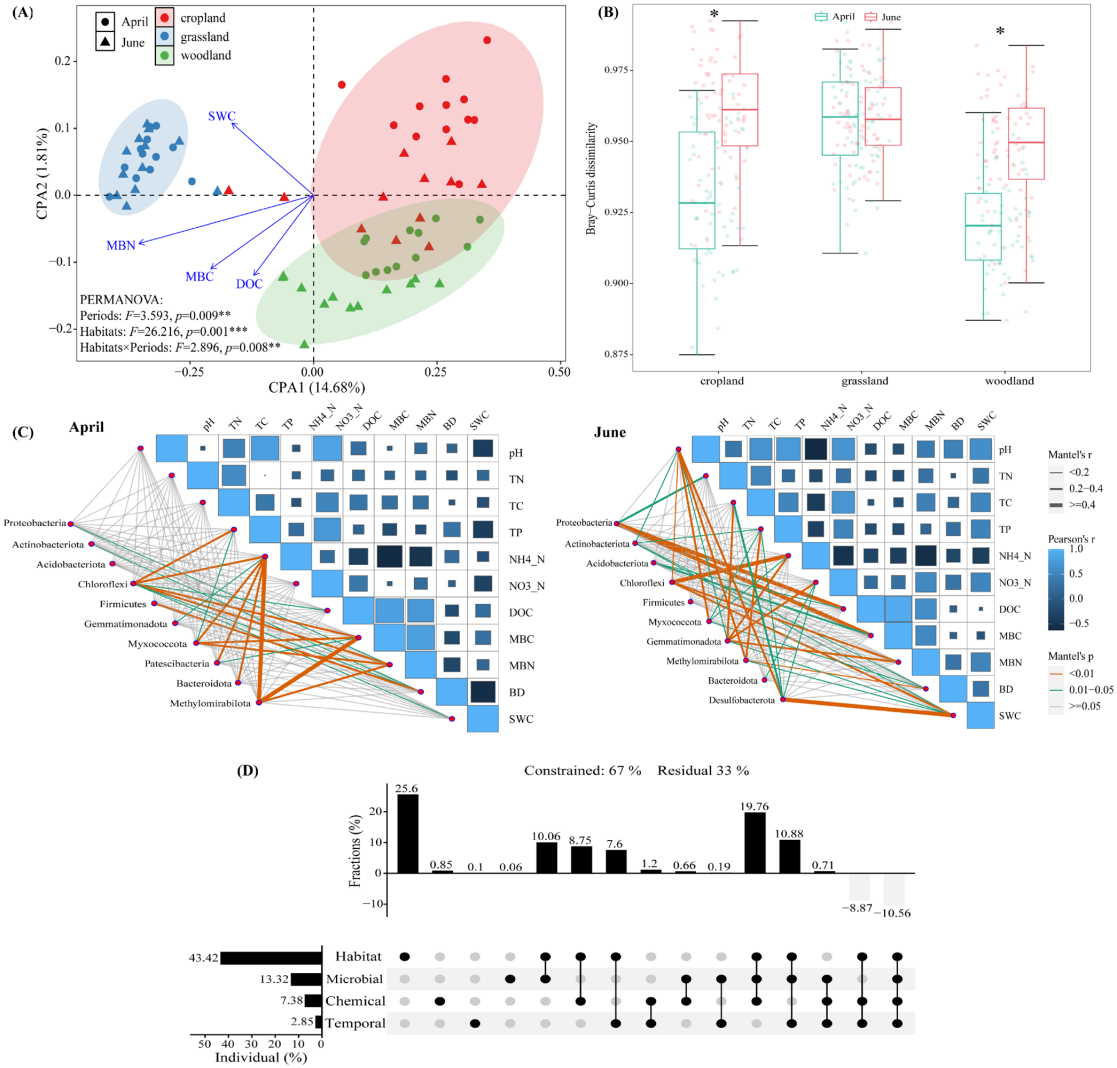
Bacterial community distributions in the three reservoir‐buffer‐strip habitats from the flooding period (April) to the dry period (June). (A) Constrained principle coordinated analysis (*CPA*), constrained to the bacterial communities on different environmental variables using weighted UniFrac distances matrix; (B) The *β* dissimilarity of the three reservoir‐buffer‐strip habitats from the flooding period (April) to the dry period (June) base on Bray–Curtis distance matrix. (C) Pairwise comparisons of soil properties of each period in the reservoir buffer strips are shown, with a color gradient denoting Spearman's correlation coefficients (* indicated significance at the 0.05 level; ** indicated significance at the 0.01 level). The top10 abundant soil bacterial taxa at the Phylum level were related to each soil property parameters of each period in the reservoir buffer strips by Mantel tests. Edge width corresponds to the Mantel's r statistic for the corresponding correlations, and edge color denotes the statistical significance based on 9999 permutations. (D) Variance partitioning analysis (*VPA*) of bacterial communities based on habitat, temporal variation, soil microbial properties, and soil chemical properties, using the weighted UniFrac distance matrix.

The habitats exhibited significantly different soil bacterial community distributions; bacterial community distribution was also affected by period and by the interaction between habitat and period (Figure 4A). PCoA was performed separately for each period and for the different habitats: soil bacterial community distribution differed significantly between the habitats within each period (Figure S2‐April, June). Except for grassland (*p* = 0.447), the other habitats exhibited significant differences in soil bacterial community distribution between the periods (Figure S2‐cropland, grassland, and woodland). Using the weighted UniFrac distance matrix, more of the variation was explained by period (April: 57.14%; June: 52.42%) than by habitat (cropland: 45.98%; grassland: 38.06%; woodland: 42.1%). Based on the Bray–Curtis dissimilarity matrix, differences in *β*‐diversity between the periods were predominantly observed for the abandoned croplands and woodlands (Figure 4B).

CPA to analyze the effects of the soil physicochemical properties on the bacterial communities revealed SWC, MBN, MBC, and DOC to be the major influencing factors (Figure 4A). We also analyzed the main physicochemical factors affecting the distribution of soil bacterial communities in different habitats and periods. The main factors affecting bacterial community distribution in the different habitats during the flooding period were BD, pH, NO_3_‐N, and NH_4_‐N (Figure S4‐April); during the dry period, they were pH, SWC, NH_4_‐N, MBN, and DOC (Figure S4‐June). The factors affecting bacterial community distribution in the different habitats varied considerably, although BD and SWC were common to all three habitats (Figure S4).; From the flooding period to the dry period, the relationship between dominant bacterial taxa and physicochemical properties was significantly enhanced, especially for high‐abundance taxa (Figure 4C). VPA revealed that habitat was the main factor affecting soil microbial community distribution in the reservoir buffer strips, with soil microbial activity being the second most important factor affecting microbial community distribution, consistent with the results of the CPA analysis (Figures 4D and S5).

### 3.3 Spatiotemporal Variation in Soil Bacterial Community Species Composition

**FIGURE 5:**
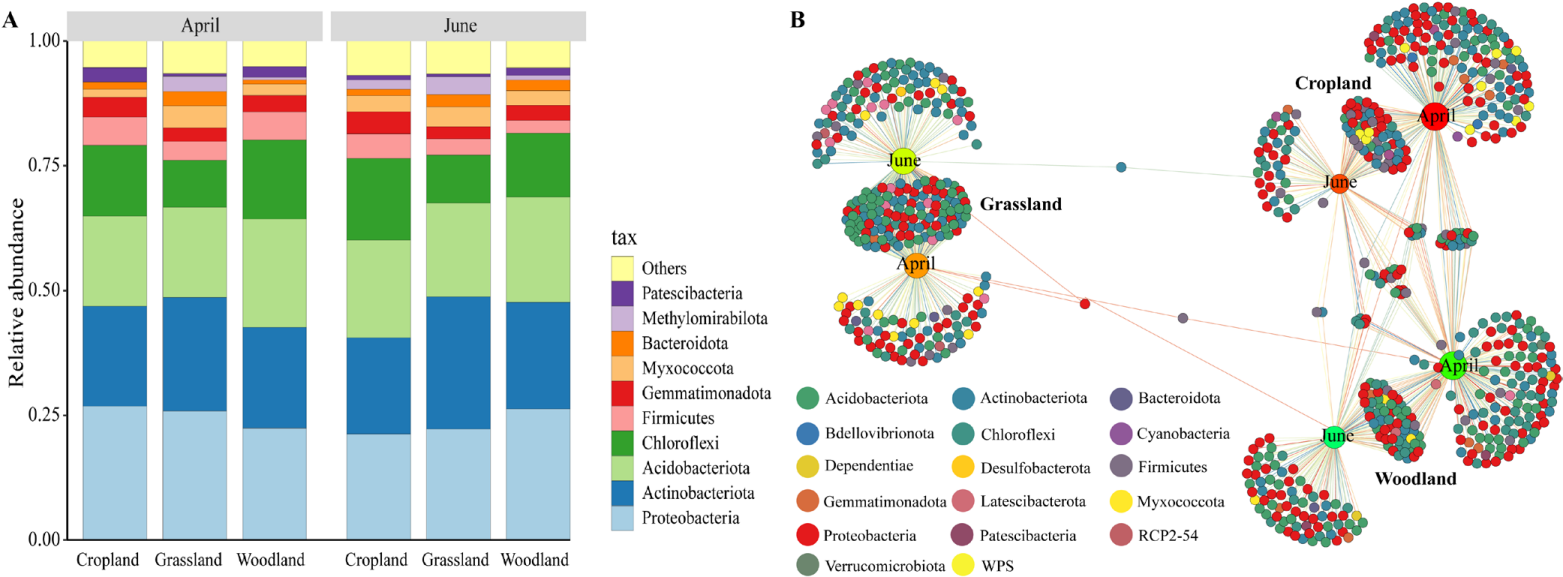
Bacterial community composition at the phylum level for the three reservoir‐buffer‐strip habitats from the flooding period (April) to the dry period (June). The bipartite networks display specific ASVs in the soil bacterial community, as determined via indicator species analysis for the different habitats. Circles represent individual bacterial ASVs that are positively and significantly associated (*p* < 0.05) with one or more of the reservoir‐buffer‐strips habitats (associations are indicated by connecting lines). ASVs are colored according to their phylum assignment.

In total, 43 phyla, 137 classes, 309 orders, 483 families, and 913 genera were identified in the reservoir buffer strips in the two periods. At the phylum level, *Proteobacteria*, *Actinobacteriota*, and *Acidobacteriota* accounted for > 15% of the total genera at each sampling site, followed by *Chloroflexi*. The top 10 most abundant phyla accounted for > 90% of the total abundance at each sampling site (Figure 5A).

We used indicator species analysis to identify individual bacterial ASVs for which the abundance varied between the habitats and periods; the results were summarized using a bipartite network (Figure 5B). Many ASVs were shared between the periods within each habitat, whereas few were shared between habitats. Significantly more shared ASVs occurred in the grassland than in the other two habitats, indicating that grassland had a stronger aggregation pattern. The numbers of ASVs specific to abandoned cropland and woodland were greater in April than in June, implying that these habitats may exhibit large differences in terms of community function between the periods (Table S1). Each habitat supports a subset of the characteristic soil microbes, and most of the bacterial communities are shared between the different periods.

These indicator species analysis results were supported by the results of the *Wilcoxon* tests of bacterial families that exhibited significant differences in abundance between periods in the same habitats (Figures S6 and S7). From the flooding period to the dry period, 19 families were enriched and 7 families were depleted in abandoned cropland; 13 families were enriched and 4 families were depleted in woodland; and only 2 families were enriched and 2 families were depleted in grassland, suggesting that the abandoned cropland and woodland experience more substantial temporal variation than the grassland.

### 3.4 Spatiotemporal Variation in Soil Bacterial Community Co‐Occurrence Networks and Ecological Processes

**FIGURE 6:**
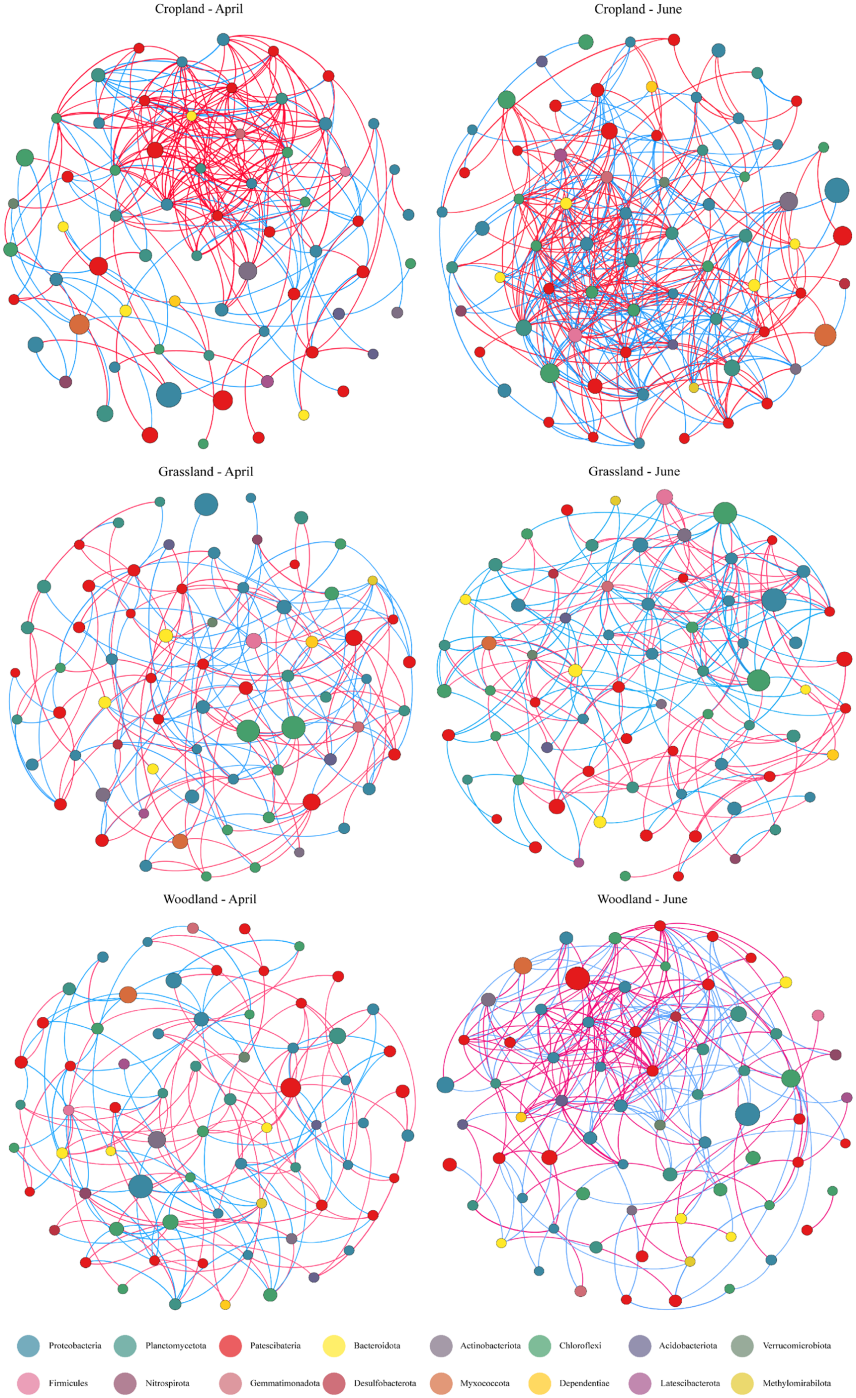
Co‐occurrence networks of the soil bacteria community of the three reservoir‐buffer‐strip habitats from the flooding period (April) to the dry period (June). The colors assigned to nodes and edges in the networks indicate different major phyla and the types of interactions, respectively. Red, positive links; blue, negative links.

From the flooding period to the dry period, soil bacterial community species interactions increased in the three habitats, as revealed by the increases in average degree, edge links, and average number of neighbors (Figure 6, Table 2); the soil bacteria exhibited more compact network topology, as revealed by the increases in edge density, degree centrality, and the cluster coefficients (with the exception of the grassland cluster coefficient, which decreased slightly); the mean distance declined; and the degree of network modularity declined, as did the proportion of negative links in the soil bacterial network for abandoned cropland (Table 2). In terms of the magnitude of the changes in network attributes between periods, abandoned cropland exhibited the most pronounced changes between the periods, followed by woodlands and grasslands.

**TABLE 2:** Co‐occurrence network topological properties for the three reservoir‐buffer‐strip habitats from the flooding period (April) to the dry period (June).

	Cropland		Grassland		Woodland	
	April	June	April	June	April	June
Node	67	72	71	71	69	70
Edge	231	335	174	202	159	222
Average degree	6.896	9.306	4.901	5.690	4.609	6.343
Negative ratio (%)	28	45	50	48	42	45
Diameter	7	6	8	7	7	7
Edge density	0.104	0.131	0.070	0.081	0.068	0.092
Clustering coefficient	0.525	0.515	0.302	0.370	0.335	0.446
Mean distance	2.920	2.601	3.182	3.032	3.373	2.973
Degree centrality	0.199	0.221	0.087	0.118	0.064	0.197
Average neighbors	8.497	11.777	5.812	6.695	5.562	8.072
Modularity	0.367	0.308	0.496	0.447	0.547	0.388

**FIGURE 7:**
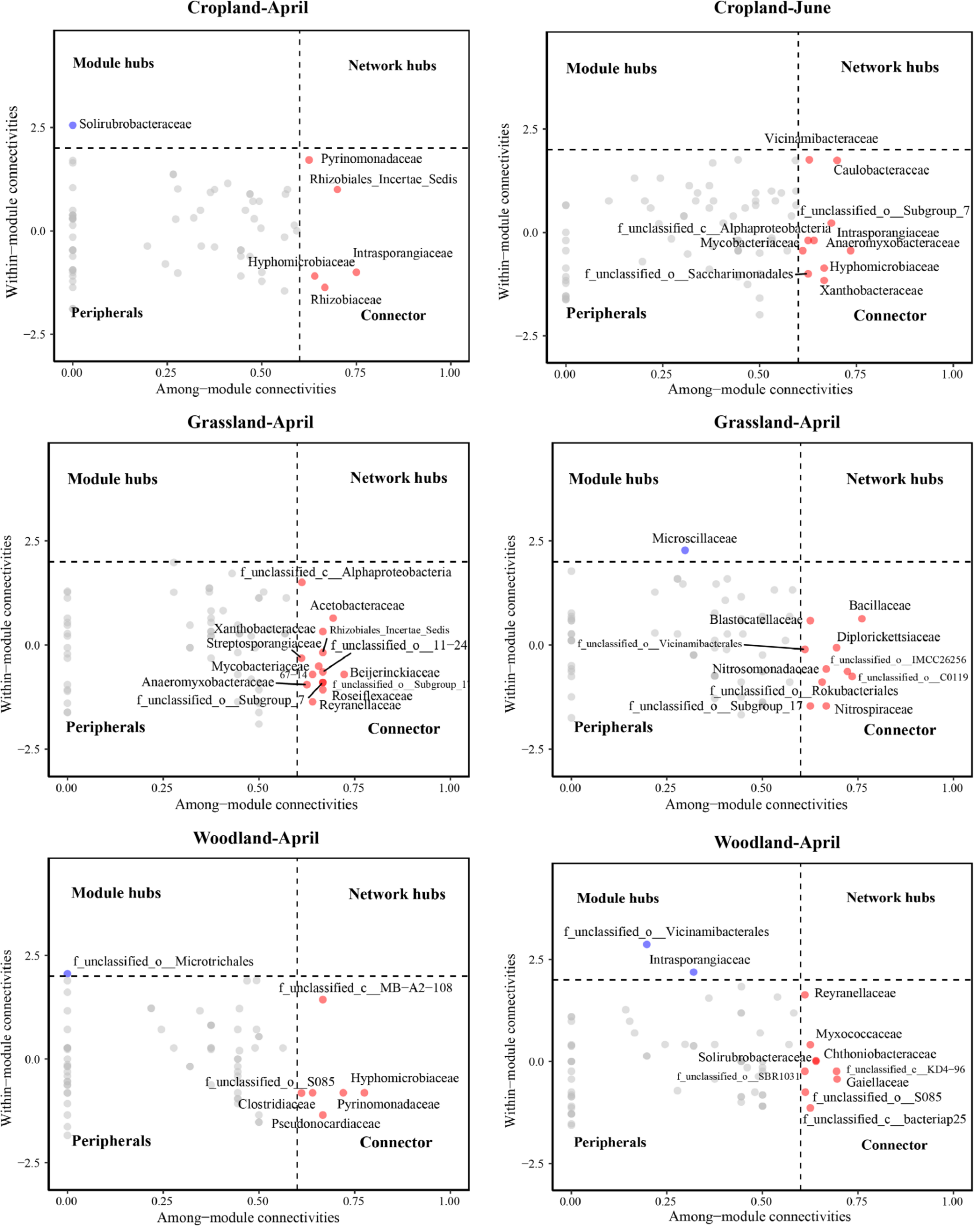
Scatter plot of *Zi* (within‐module connectivity) and *Pi* (among‐module connectivity). The threshold values of *Zi* and *Pi* for categorizing ASVs were 2.5 and 0.62, respectively.

Relative to the flooding period, more keystone species were present in the abandoned cropland and woodland during the dry period. In the abandoned cropland, 1 module hub and 5 connectors were identified in April, whereas 10 connectors were identified in June; in grassland, 13 connectors were identified in April, and 1 module hub and 11 connectors were identified in June; and in woodland, 1 module hub and 6 connectors were identified in April, and 2 module hubs and 11 connectors were identified in June (Figure 7).

**FIGURE 8:**
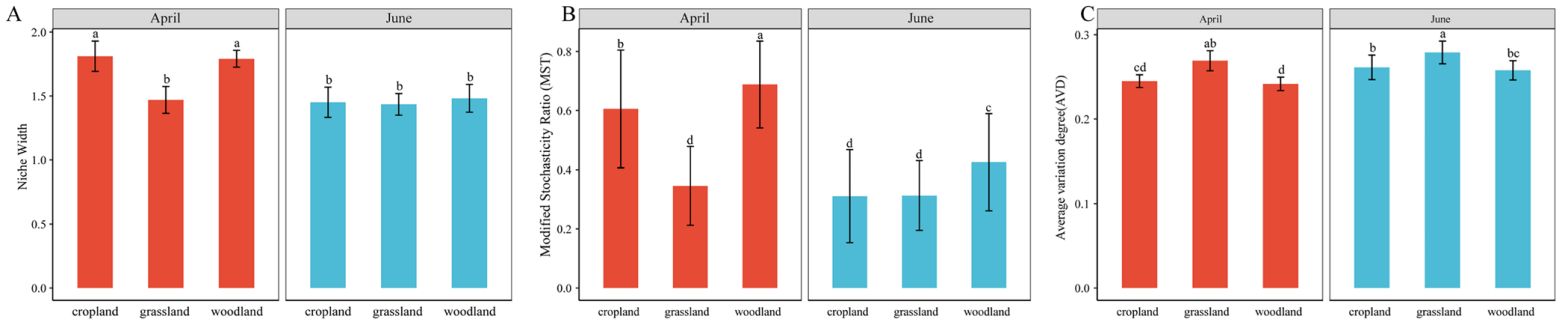
Bacterial community niche width, modified stochasticity ratio (MST), and average variation degree (AVD) for the three reservoir‐buffer‐strip habitats from the flooding period (April) to the dry period (June). A higher MST indicates that the community assembly is more stochastic; the smaller the AVD value, the more stable the microbial community is.

From the flooding period to the dry period, the niche widths of the soil bacterial communities decreased significantly for abandoned cropland and woodland, and the community assembly processes changed from stochastic to deterministic (MST > 0.5 in April; MST < 0.5 in June). The AVD of grassland was significantly greater than that of abandoned cropland and woodland in both periods, indicating that grassland had lower bacterial community stability. From the flooding period to the dry period, the AVD values of abandoned cropland and woodland increased significantly, whereas that of grassland did not, indicating that desiccation reduced bacterial community stability in these two habitats (Figure 8).

**FIGURE 9:**
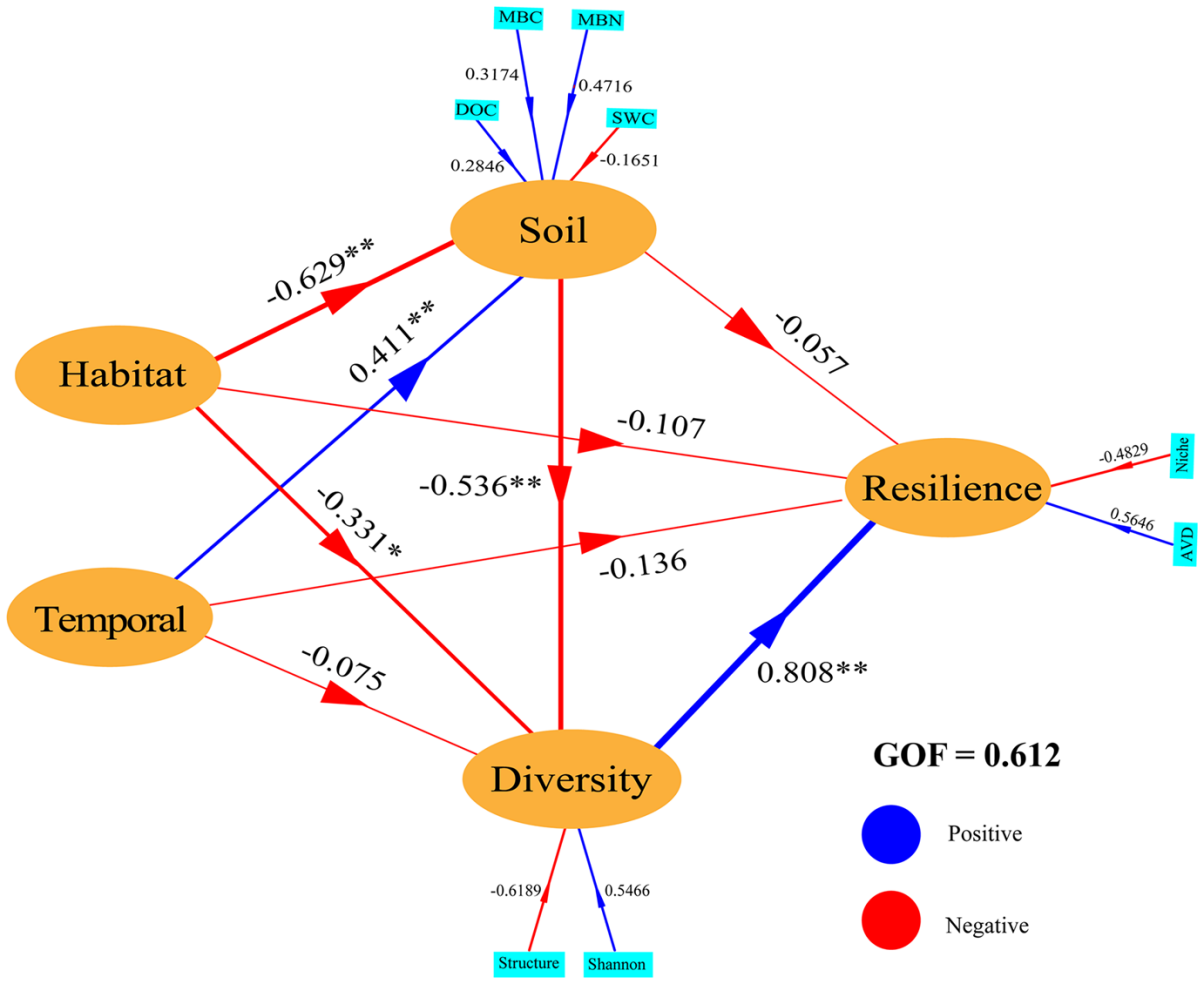
Partial Least Squares Structural equation modeling (PLS‐SEM) demonstrates the potential regulatory mechanisms of habitat and temporal on microbial community resistance. The arrow width shows the strength of the standardized path coefficient. The blue lines show positive path coefficients, and the red lines show negative path coefficients. “*” means significant at the 0.05 level, “**” means significant at the 0.001 level; GOF (goodness of fit).

PLS‐SEM analysis further revealed the potential mechanisms of habitat and temporal on the resilience capacity of bacterial communities. Only bacterial diversity had direct effects on bacterial community resilience capacity. Habitat can directly or indirect (through soil properties) regulate bacterial diversity, which in turn affects bacterial community resilience capacity, while temporal variation only had directly effects on soil properties (Figure 9).

**FIGURE 10:**
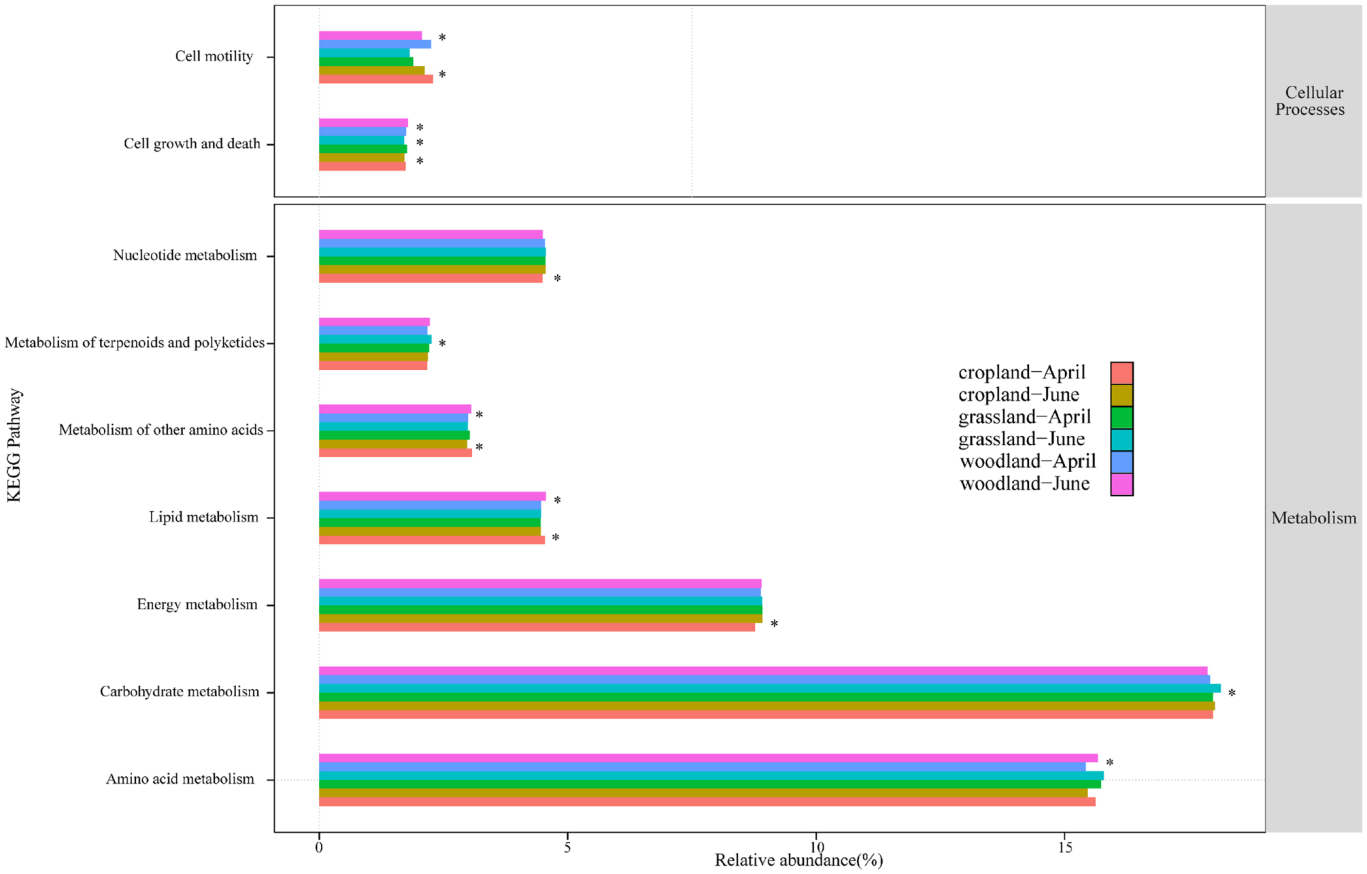
Bacterial community ecological function in the three reservoir‐buffer‐strip habitats from the flooding period (April) to the dry period (June). The asterisks (*) represent significant differences in the corresponding ecological functions between different periods.

The results of the PICRUST2 prediction analysis revealed that bacterial community ecological function was primarily related to the metabolism of carbohydrates, amino acids, and energy (Figure 10). We observed specific responses in microbial community function between periods in different habitats; Amino acid metabolism changed significantly between different periods in woodland (*p* = 0.120); Energy metabolism and nucleotide metabolism changed significant between different periods in abandoned cropland (*p* < 0.001); Carbohydrates metabolism and terpenoids and polyketides changed significantly between different periods in grassland (*p* = 0.038; *p* = 0.024); Cell growth and death changed significantly between different periods in all three habitats (abandoned cropland, *p* = 0.028; grassland, *p* = 0.017; woodland, *p* = 0.017). Cell motility changed significantly between different periods in abandoned cropland and woodland (*p* = 0.004; *p* = 0.001). This indicates that the response of soil microorganisms to environmental changes in reservoir buffer strips is regulated primarily by differences in bacterial community ecological function between habitats.

## 4 Discussion

Numerous studies have reported the responses of bacterial community composition to habitat and temporal variation (Pereira e Silva et al. 2012; Gonzalez et al. 2012; Wei et al. 2022). However, it is difficult to strictly separate these factors because habitat itself may be characterized by a combination of various environmental factors. For instance, Wang et al. (2012) found that land use alone could explain ca. 30% of the variation in soil organic matter in reservoir buffer zones, whereas the inclusion of environmental factors explained only ca. 1% of the variation. Therefore, elucidating the mechanisms of temporal and habitat effects on bacterial communities can help deepen our understanding of the spatiotemporal variability of soil microbiological processes in reservoir buffer strips. Our current findings provide strong support for the existence of different mechanisms of response to temporal variation in bacterial communities in different reservoir‐buffer‐strip habitats, which are mainly reflected in changes in bacterial community species composition and function. Consistent with our hypotheses, we found that the primary factor driving the spatial and temporal variation of bacterial communities in reservoir buffer strips was the resilience capacity of different habitats to environmental disturbance.

### 4.1 Spatiotemporal Variation in Bacterial Community Species Composition and Diversity

Temporal and spatial variability exerts scale‐dependent effects on bacterial communities (Zhang et al. 2020). At continental scales, the importance of geospatial variation over temporal variation has been widely demonstrated (Pereira e Silva et al. 2012; Xun et al. 2021; Wei et al. 2022). In contrast, at the regional scale, bacterial community composition and function do not respond uniformly to temporal variation, owing to the unique geochemical cycling processes in different habitats (Samaritani et al. 2017; Barboza et al. 2018; Lan et al. 2018; Xiang et al. 2021; Chen et al. 2023). Here, we observed significant effects of both temporal variation and habitat on bacterial community diversity (Figure 3). The *α*‐diversity indices of the habitats tended to increase from the flooding period to the dry period, although insignificantly; previous studies have observed insignificant changes in *α*‐diversity in disturbed ecosystems (Heděnec et al. 2018; Xu et al. 2021; Yu et al. 2022; Jiajia et al. 2023) and have explained this in terms of the location of the sampling site (Yan et al. 2023), historical legacy effects (Allison 2023), and environmental change drivers (Campisano et al. 2017; Schimel 2018). Bacterial community *β*‐diversity differed significantly between the three habitats and varied between the periods within each habitat (primarily within the abandoned cropland and woodland). This indicates that habitat can affect the response patterns of soil bacterial communities to spatiotemporal variation in the reservoir buffer strips. This is consistent with the seasonal changes in bacterial communities in rubber and rainforest plantations from the dry to rainy season on the tropical Hainan Island (Wei et al. 2022).

Temporal variation in terrestrial ecosystems primarily alters soil bacterial community abundance rather than species composition (Caporaso et al. 2012; Barboza et al. 2018). In wetland ecosystems, in contrast, where there is more emphasis on the importance of habitat in shaping microbial communities, subtle environmental heterogeneity is sufficient to drive changes in bacterial community *β*‐diversity (Lowell et al. 2009; Wang et al. 2021; An et al. 2023). The present study provides compelling evidence that soil bacterial communities in reservoir buffer strips have a dual response mechanism whereby species composition and function respond to temporal variation, as illustrated in the bipartite networks, in which almost no ASVs are shared between habitats (Figure 5B). This suggests that there may be great differences in community function between habitats (in terms of plant recruitment) (Barboza et al. 2018). Here, within each habitat, many of the ASVs were unique to each period, while there were also many ASVs shared between the periods. This is potentially because changes between the periods in soil moisture and climatic conditions in the reservoir buffer strips lead to the death, dormancy, or resurgence of some bacterial taxa (Fierer 2017; Schimel 2018; Allison 2023). Notably, these unique bacterial taxa were affiliated predominantly with several of the more abundant phyla (Table S1). We speculate that the previous emphasis on changes in microbial community species abundance may have obscured the importance of species composition.

### 4.2 Factors Driving Spatiotemporal Variation in the Bacterial Community

Soil properties such as pH, temperature, soil organic carbon, and oxygen partial pressure have emerged as key drivers of microbiome structure and microbial activity (Campisano et al. 2017; Fierer 2017; Philippot et al. 2023). Here, the main factors affecting the soil bacterial community distribution were not consistent across the different scales of consideration, reflecting the complex spatiotemporal variation in soil properties in the reservoir buffer strips. The explanatory power of soil properties in terms of the bacterial community differed significantly between habitats and periods. Soil properties explained the changes in bacterial community composition between different habitats during the same period (April: 42.17%, June: 32.54%), whereas they were less effective in explaining these differences between periods within habitats (Figure S2). In addition, the weighted UniFrac distance matrix explained significantly more of the variation in bacterial community distribution than the Bray‐Curtis distance matrix (Figures 4A and S3); this further suggests the importance of changes in bacterial phylogeny over changes in species abundance in reservoir buffer strips (An et al. 2023).

From the perspective of the dominant factors, the main soil parameters affecting the changes in the bacterial community between the different periods within the same habitat were more consistent (BD and SWC), indicating that differences between the periods in soil moisture status and physical properties were the main drivers of changes in the bacterial community in the reservoir buffer strip (Figures S4). However, the primary soil‐related factors driving the differences in the bacterial communities between habitats within the same period varied considerably; this may be related to the unique responses of habitats to environmental changes. For example, woodland buffer strips are effective in inhibiting denitrification under flooding conditions, accelerating the accumulation of NO_3_‐N (Yan et al. 2023). On the whole, the primary factors driving the changes in the soil bacterial community were SWC, MBC, MBN, and DOC, indicating that soil moisture status and microbial activity were the primary factors determining bacterial community spatiotemporal variation in the reservoir buffer strips. This is consistent with prior findings for riparian wetland ecosystems (Yao et al. 2019), that land‐use type regulates carbon and nitrogen nutrient turnover via microbial stoichiometry. This combination of results is not unexpected; for instance, Xiang et al. (2021) argued that different processes may dominate microbial systems at different spatial, temporal, and phylogenetic scales.

### 4.3 Spatiotemporal Variation in Soil Bacterial Community Ecological Processes

Although limited information is available on the connections between microbial ecology and ecosystem properties, co‐occurrence networks can provide valuable information in this regard (Zhu et al. 2022; Guseva et al. 2022). Here, the bacterial networks in three habitats responded to temporal variation, in accordance with the ecological consequences of environmental filtration driven by factors such as the rapid drying of the reservoir buffer strips during the experimental period and seasonal warming (De Vries et al. 2018; Yuan et al. 2021; Pescador et al. 2022). Soil bacterial community network complexity in all three habitats was greater in the dry period (Figure 6). This is consistent with the results of Yuan et al. (2021), who observed the effects of climate warming on the spatiotemporal variability of soil bacterial communities in grassland ecosystems. Furthermore, the transition from the flooding period to the dry period resulted in a more compact and aggregate bacterial co‐occurrence pattern; this enables the effects of perturbations generated by temporal variation to be rapidly distributed throughout the network, making the entire system more efficient (Morriën et al. 2017; Wang et al. 2022). Bacterial community assembly in the three habitats was ultimately characterized primarily by deterministic processes, with a reduction in community stability, reflecting the filtering of soil bacterial community by the drastic changes in the environmental conditions of the reservoir buffer zone (Yang et al. 2019).

We place more emphasis on the fact that the resilience capacity of the habitats drives the differences in the responses of bacterial ecological processes to temporal variation. For example, the transition from the flooding period to the drying period caused a significant increase (from 28% to 45%) in the rate of negative linkages within the abandoned cropland soil bacterial networks; this suggests that temporal variation exacerbates competition for ecological niches among the bacterial taxa in abandoned croplands in reservoir buffer strips (Gao et al. 2022; Zhu et al. 2022); With the transition from the flooding period to the dry period, bacterial community assembly processes in the abandoned cropland and woodland shifted from being stochastic to deterministic; this is potentially related to the reduced resource availability, owing to the loss of a large amount of organic matter as the water level drops during the dry period (Liu et al. 2021; Ran et al. 2021). Reduced resource availability causes microbial community assembly processes to shift from stochastic to deterministic (Yan et al. 2022);

With the transition from the flooding period to the dry period, the number of core bacterial taxa increased significantly in the abandoned cropland and woodland (Table S2). Typically, the presence of more key taxa indicates higher stability of the network (Coyte et al. 2015), suggesting that temporal variation promotes network stability of bacterial communities in abandoned croplands and woodlands. These core ASVs are affiliated with high‐abundance taxa such as *Proteobacteriota*, *Actinobacteriota*, and *Acidobacteriota*, which are important in ecosystem transformation and soil nutrient cycling and can adapt to drastic changes in oxidation–reduction environmental conditions (Ansari et al. 2023; Chen et al. 2024). We also observed that core ASVs affiliated with *Chloroflexi* were only found in grassland and woodland, where these taxa can survive in nutrient‐poor soils and play a pivotal role in soil ecosystems through interactions with plant roots (Qu et al. 2020; Wang et al. 2023). From the flooding period to the dry period, we also observed some core ASVs affiliated with low‐abundance taxa only present in the dry period, which may implicate the importance of rare species in drastically changed ecosystems (Tian et al. 2024). Such as *Patescibacteria* in abandoned cropland, often reported to appear in polluted environments (Tian et al. 2020; Fujii et al. 2022); *Methylomirabilota* and *Nitrospirota* in grassland are two phyla that are known to participate in the carbon and nitrogen cycle process in riparian ecosystems (Li, Siddique et al. 2022; Zhang, Zhang et al. 2023; Tian et al. 2024); and *Verrucomicrobiota* in woodland has been proven to play a major role in the degradation of plant‐derived organic matter (Rakitin et al. 2024).

Together, these results suggest that bacterial network complexity and stability in these three habitats, arising from temporal variability, may strongly influence the functional structure of the bacterial community and thus affect ecosystem functioning processes.

### 4.4 Mechanisms Underlying the Different Responses of Habitats to Spatiotemporal Variation

It is well known that soil bacterial communities in different habitats regulate ecosystem function via their tolerance and resilience to environmental changes (Freedman et al. 2015; Karimi et al. 2017). Yu et al. (2023) found that spatiotemporal patterns of soil bacterial communities in floodplain ecosystems were not consistent when examined using different agronomic measures. Here, however, we observed consistent patterns of temporal variation in the soil bacterial community in terms of community diversity, the dominant driving factors, and the ecological processes in the different habitats, reflecting the deterministic shaping of bacterial communities by specific environmental changes in this reservoir buffer strip. Further, there were distinct differences in the magnitude and intrinsic mechanisms of bacterial community changes and ecology processes in the three habitats. For example, the soil bacterial *β*‐diversity, network structure, and ecological processes of abandoned cropland and woodland differed between the periods, with abandoned cropland changing the most, while grassland showed relatively small differences. This may be related to the resilience capacity of these habitats to environmental change. Grasslands are characterized primarily by perennial herbaceous vegetation with well‐developed fine root structures, improving their soil and water retention and underground nutrient input; this may be the primary factor supporting the stability of their soil bacterial communities (Hunt and Ward 2015). In contrast, abandoned croplands are characterized primarily by annual herbaceous plants and exhibit high turnover owing to water level fluctuation, causing drastic changes in their bacterial community species composition and function (Zhang, O'Connor et al. 2021). In conclusion, the resilience capacity of the habitats in the reservoir buffer strips to the environment mediated the response of their soil bacterial communities to spatiotemporal variation.

This study did ignore soil microbial kinetics originating from time‐dependent factors such as soil moisture, temperature, nutrient levels, and vegetation biomass (Campisano et al. 2017; Taketani et al. 2017) and may have overemphasized the deterministic process whereby habitat shapes the bacterial communities. This study observed only the variation patterns of bacterial communities from a period of flooding to a dry period of the reservoir buffer strips in the same years and lacked dynamic monitoring of inter‐annual changes in bacterial communities during water‐level fluctuations in a long time series.

## 5 Conclusion

The complex hydrological situation of the reservoir buffer zone made it difficult to accurately distinguish the contribution of temporal and spatial on microbiome changes (Regan et al. 2014). This study aimed to explore the spatiotemporal patterns of bacterial communities in different reservoir‐buffer‐strips habitats located in the same hydraulic gradient. The results demonstrated that habitat is more important than temporal variation in shaping the spatiotemporal dynamics of bacterial communities in reservoir buffer zones, which is in contrast with the previous study (Samaritani et al. 2017).

This study underscored the significance of the species composition and functionality of different reservoir‐buffer‐strips habitats on the spatiotemporal variability of bacterial communities at the level of Amplicon Sequence Variants (ASVs), thereby enriching the conventional understanding of abundance changes due to the merging of species subgroups during microbial bioinformatics analyses. Temporal variation affects bacterial communities by altering the abundance of shared species and the resurgence or extinction of unique taxa within identical habitats. Habitats adapt to changing reservoir bank environments by regulating the composition and function of their soil bacterial communities, which may be closely related to the characteristics of the vegetation growing in them, implying the importance of vegetation management in the environmental maintenance of the reservoirs buffer zones. The traditional approach to reservoir buffer zone management advocates the modification of local abiotic variables to increase biodiversity (Zhang, Li et al. 2023). We should also pay attention to the important role of habitat types in maintaining the diversity and stability of bacterial communities, especially the allocation ratios of different habitat vegetation types and landscape diversity, in order to improve the integrated ecological benefits of the reservoir buffer zone from a multiscale perspective.

This study will help to elucidate the response of soil bacterial communities of different habitats in the reservoir buffer zone to temporal variation and will provide a scientific basis for the management of the reservoir shoreline from the perspective of microbial ecology.

## Author Contributions

**Tengfei Yan:** data curation (equal), investigation (equal), writing – original draft (equal). **Zhengxin Wang:** data curation (equal), investigation (equal), methodology (equal). **Zhen Wang:** data curation (equal), data curation (equal), investigation (equal), investigation (equal). **Yong Qin:** investigation (equal), methodology (equal). **Zheng Wang:** data curation (equal), writing – review and editing (equal). **Songwei Li:** conceptualization (equal), supervision (equal), writing – review and editing (equal).

## Conflicts of Interest

The authors declare no conflicts of interest.

## Supporting information


Data S1.

## Data Availability

Data have been archived on publicly accessible repositories: 16S rRNA sequences are available from the National Microbiology Data Center (NMDC) Sequence Read Archive with BioProject accession number NMDC10018801. Soil physicochemical properties data and R codes available are from the Dryad Digital Repository. http://datadryad.org/stash/share/TW‐jDYZF_ZyiTB5GjPCSrolQOsVkrLP48cdyfSpqrWg.
