# Supplementary material for: Temporal Variation in Bacterial Community Characteristics Shaped by Habitat in a Reservoir Buffer Strip in China
Source: Ecol Evol. 2025 Mar 7;15(3):e70957. doi: 10.1002/ece3.70957 (PMC11885952; doi:10.1002/ece3.70957)
Supplement: Supplementary file 1 — Data S1. [file ECE3-15-e70957-s001.doc]

**Supplemental Material**

**Temporal variation in bacterial community characteristics shaped by habitat in a reservoir buffer strip in China**

Tengfei Yan^1,4^, Zhengxin Wang^1,2^, Zhen Wang^1^, Yong Qin^1^, Zheng Wang^3^, Songwei Li^2^*

^1^ Xinyang Huai River Catchment Riparian Zone Carbon Neutralization Engineering Technology, Xinyang Agriculture and Forestry University, Xinyang 464000, P.R.China

^2^ Henan Province Engineering Research Center of Biological Pesticide & Fertilizer Development and Synergistic Application, Henan Institute of Science and Technology, Xinxiang 453000, P.R.China

^3^ School of Landscape Architecture and Art, Henan Agriculture University, Zhengzhou 450000, P.R.China

^4^ Xinyang Ecological Research Institute, Xinyang 464000, P.R.China

^*^Corresponding author.

Email addresses: [lear9999@163.com](mailto:lear9999@163.com) (S. Li).


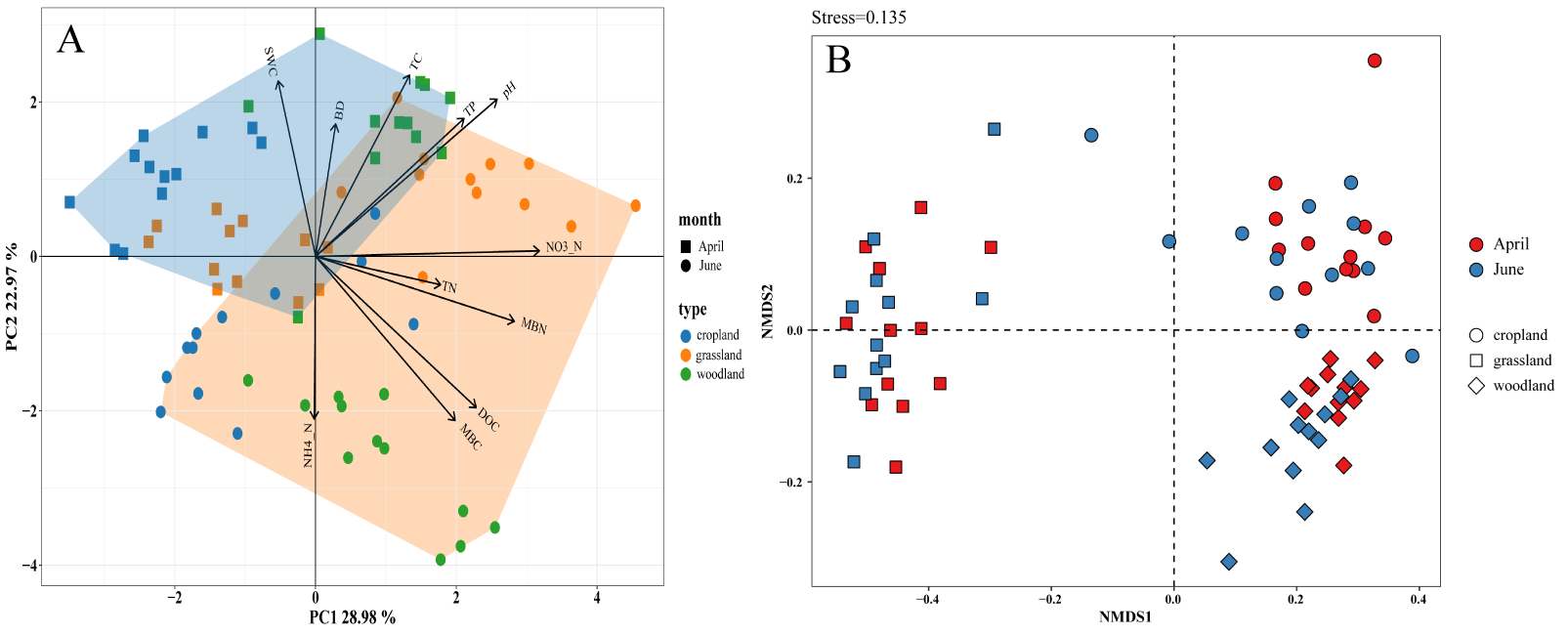


Fig. S1. (A) Principal Component Analysis (*PCA*) of soil properties of three habitats in the reservoir buffer strips from the flooding period (April) to the dry period (June).; (B) Non-metric multidimensional scaling (*NMDS*) of soil bacterial community of three habitats in the reservoir buffer strips from the flooding period (April) to the dry period (June).


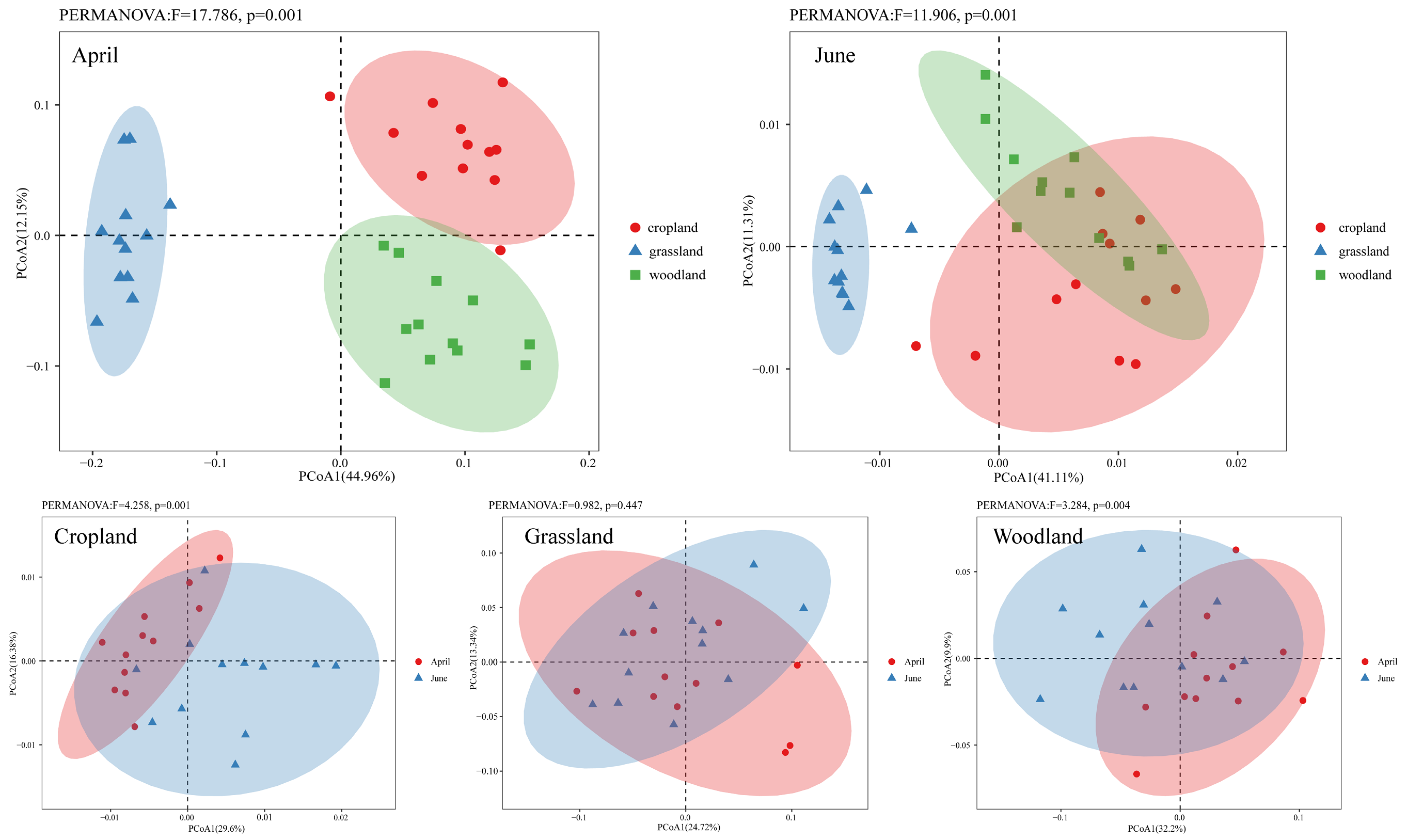


Fig. S2. The principal coordinates analysis (*PcoA*) based on Weighted Unifrac distances matrix of bacterial communities for different periods and habitats in the reservoir buffer strips, respectively.


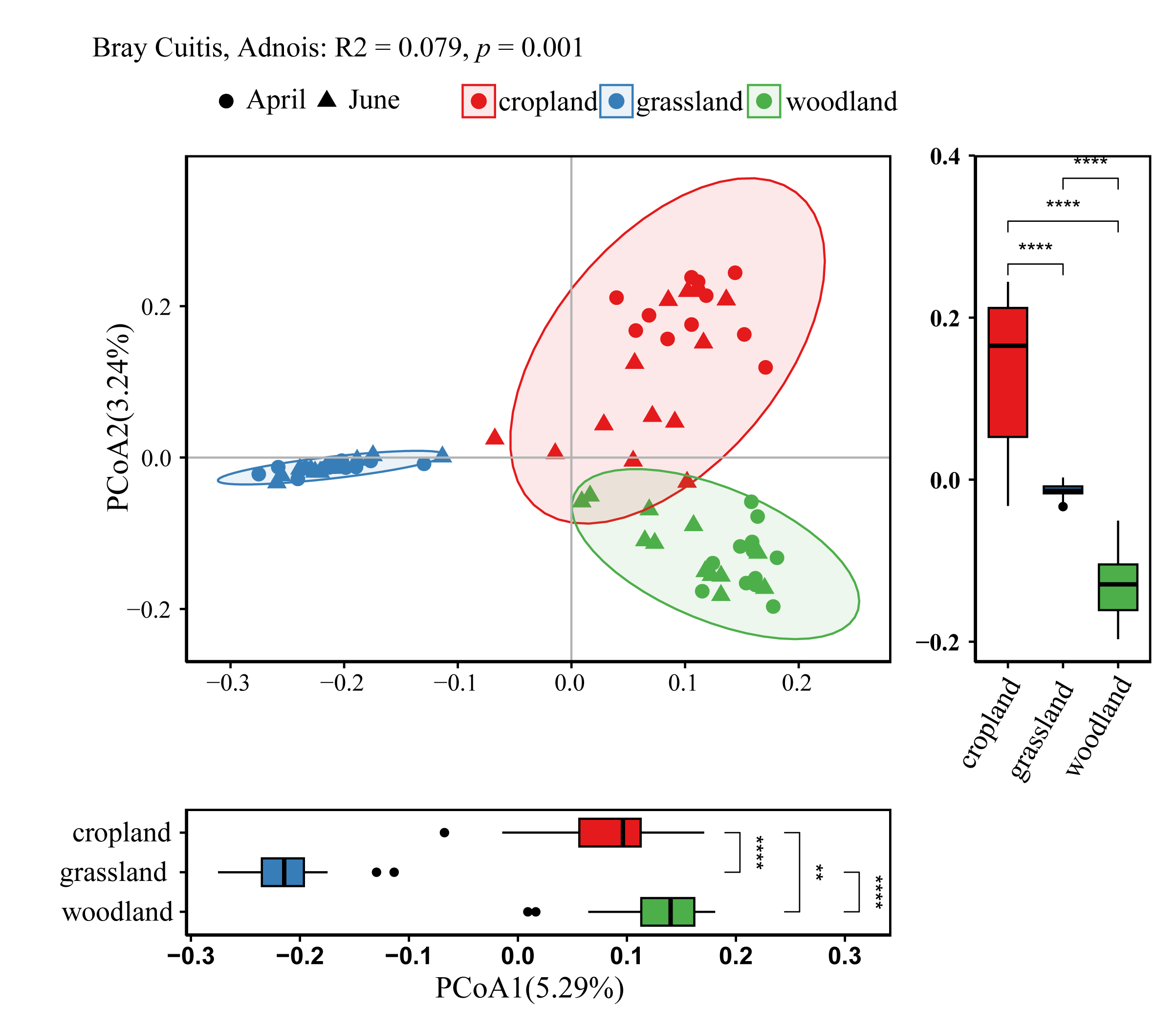


Fig. S3. The principal coordinates analysis (*PcoA*) analysis constrained to bacterial communities based on Bray Curist distances matrix for different habitats in the reservoir buffer strips from the flooding period (April) to the dry period (June).


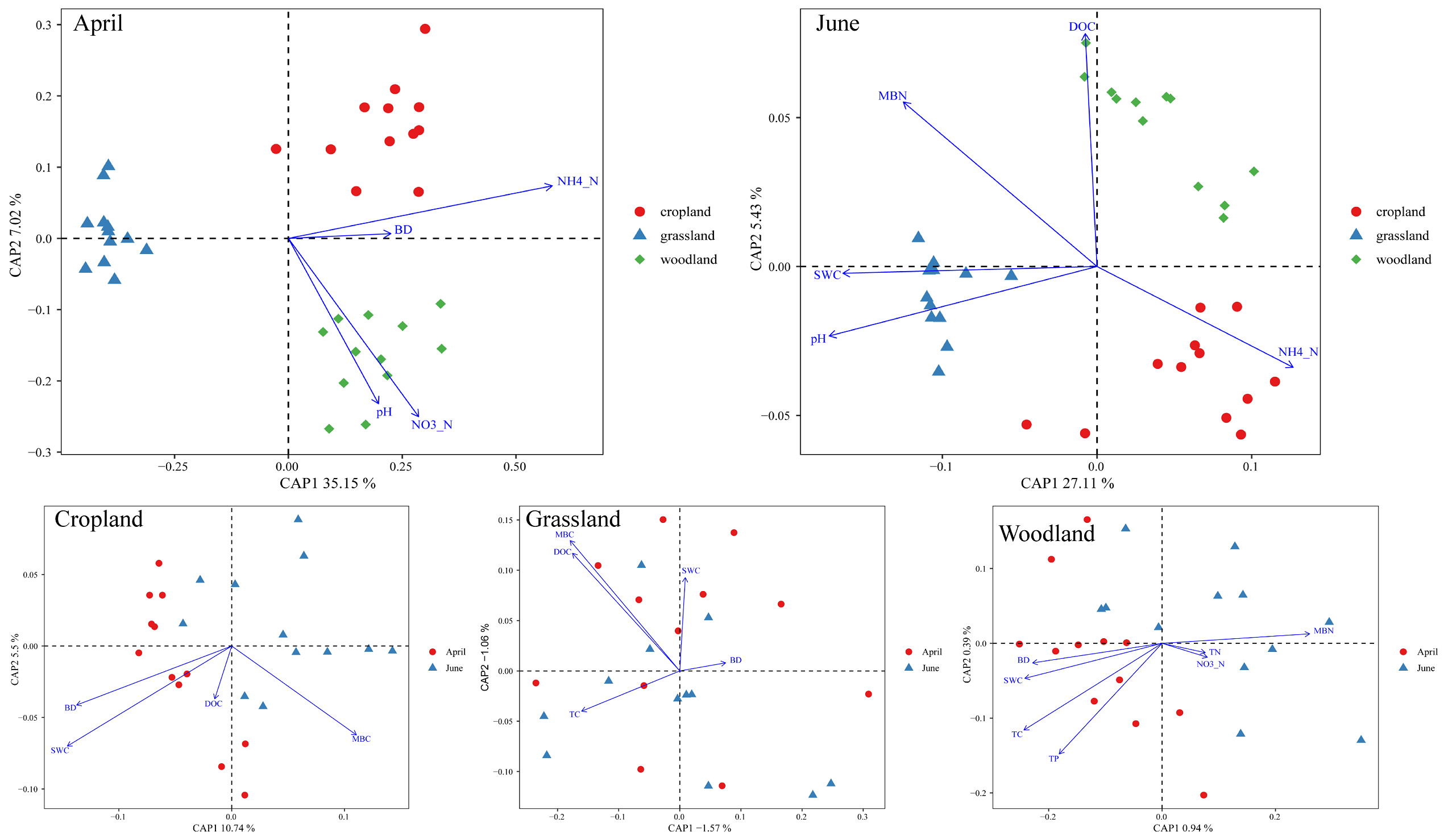


Fig. S4. The Constrained principle coordinated analyses (*CPAs*) analysis constrained to bacterial communities based on Weighted Unifrac distances matrix for different periods and habitats in the reservoir buffer strips, respectively.


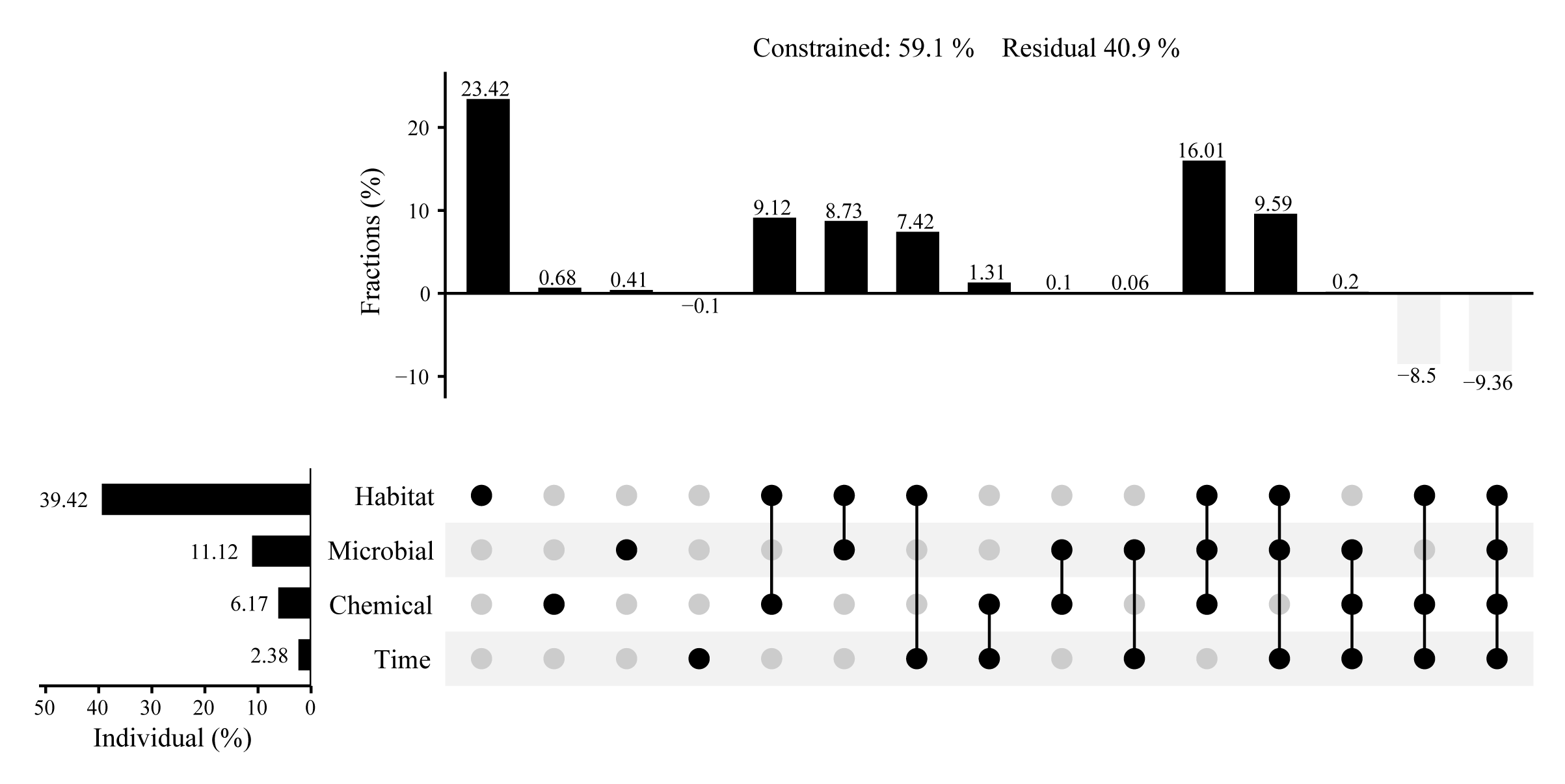


Fig. S5. Variance partitioning analysis (*VPA*) of bacterial communities based on the habitat, temporal, soil microbial properties, and soil chemical properties variables by using unweighted Unifrac distance matrix.


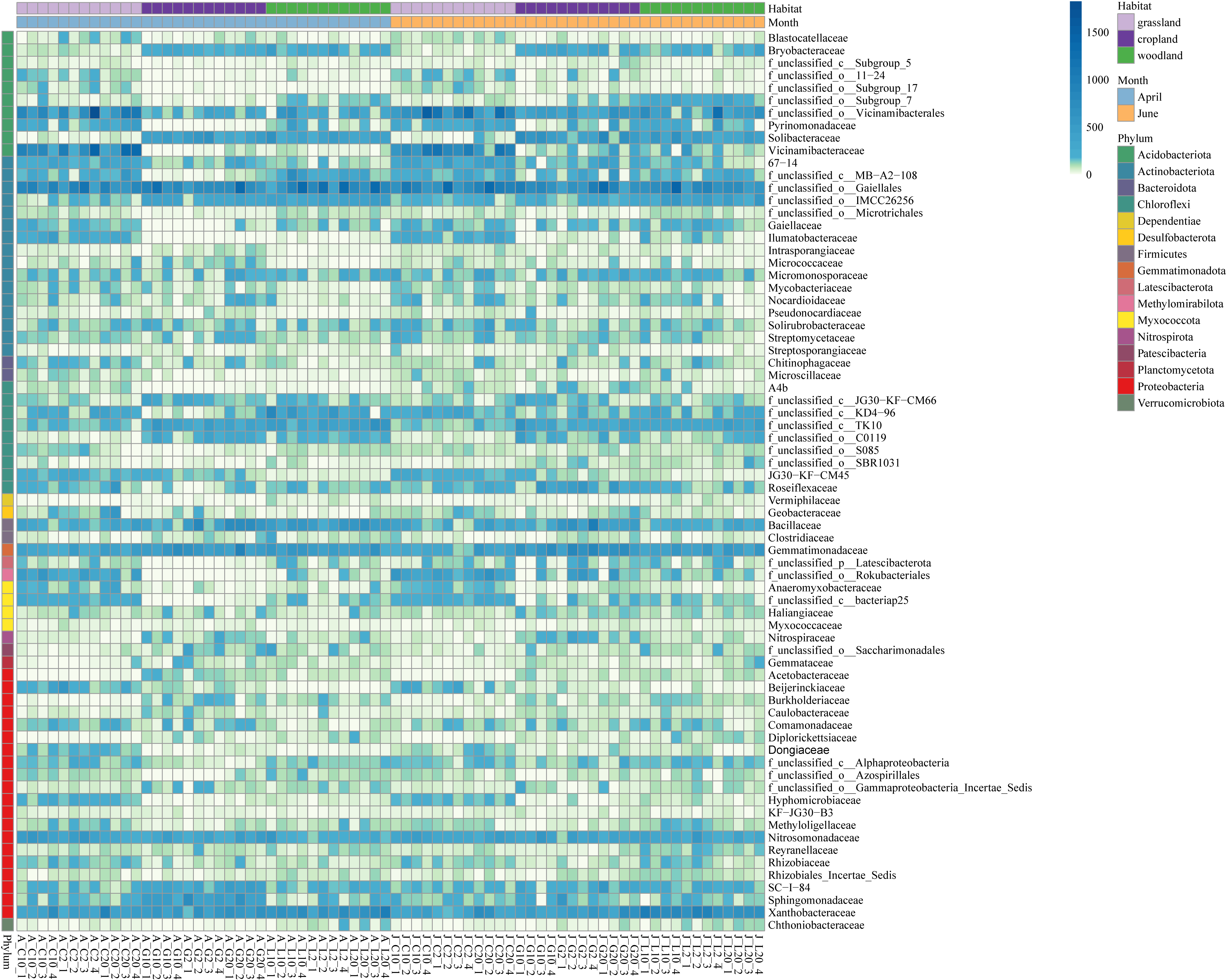


Fig. S6. Correlation heat map display the variation patterns of soil bacterial community at Family level in three habitats of the reservoir buffer strips from the flooding period (April) to dry period (June).


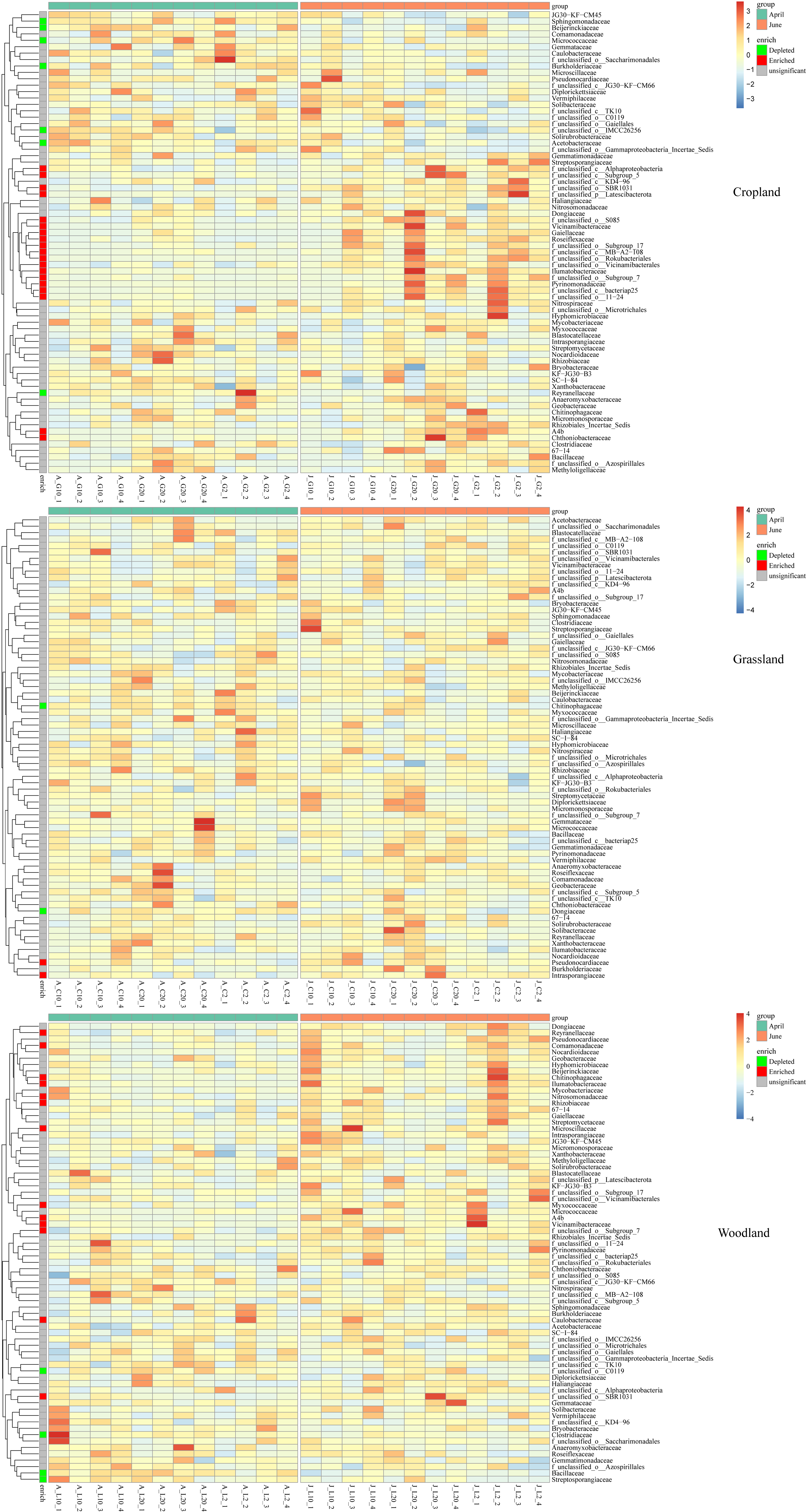


Fig. S7. Correlation heat map display the *Wilcox test* results of soil bacterial community at Family level between different period in each habitat in the reservoir buffer strips.

Table S1 The number and affiliation of shared and individual bacterial taxa (significant changed) between different periods in each habitat of reservoir buffer strips.

| Land-use | Month | Phylum | Count | Total count |
| --- | --- | --- | --- | --- |
| Cropland | April | *Proteobacteria* | 44 | 128 |
|  |  | *Actinobacteriota* | 25 |  |
|  |  | *Acidobacteriota* | 24 |  |
|  |  | *Chloroflexi* | 13 |  |
|  |  | *Firmicutes* | 10 |  |
|  |  | *WPS-2* | 7 |  |
|  |  | *Gemmatimonadota* | 4 |  |
|  |  | *Nitrospirota* | 1 |  |
|  | June | *Proteobacteria* | 16 | 34 |
|  |  | *Proteobacteria* | 7 |  |
|  |  | *Actinobacteriota* | 4 |  |
|  |  | *Firmicutes* | 3 |  |
|  |  | *Acidobacteriota* | 2 |  |
|  |  | *Gemmatimonadota* | 2 |  |
|  | Share | *Proteobacteria* | 21 | 56 |
|  |  | *Actinobacteriota* | 14 |  |
|  |  | *Firmicutes* | 11 |  |
|  |  | *Acidobacteriota* | 5 |  |
|  |  | *Chloroflexi* | 3 |  |
|  |  | *Nitrospirota* | 1 |  |
|  |  | *WPS-2* | 1 |  |
| Grassland | April | *Proteobacteria* | 20 | 51 |
|  |  | *Actinobacteriota* | 10 |  |
|  |  | *Acidobacteriota* | 8 |  |
|  |  | *Firmicutes* | 4 |  |
|  |  | *Chloroflexi* | 3 |  |
|  |  | *Methylomirabilota* | 3 |  |
|  |  | *Myxococcota* | 3 |  |
|  | June | *Actinobacteriota* | 17 | 48 |
|  |  | *Acidobacteriota* | 13 |  |
|  |  | *Proteobacteria* | 9 |  |
|  |  | *Chloroflexi* | 3 |  |
|  |  | *Methylomirabilota* | 3 |  |
|  |  | *Firmicutes* | 2 |  |
|  |  | *Myxococcota* | 1 |  |
|  | Share | *Acidobacteriota* | 33 |  |
|  |  | *Proteobacteria* | 28 | 105 |
|  |  | *Actinobacteriota* | 27 |  |
|  |  | *Chloroflexi* | 10 |  |
|  |  | *Methylomirabilota* | 5 |  |
|  |  | *Gemmatimonadota* | 1 |  |
|  |  | *Myxococcota* | 1 |  |
| Woodland | April | *Proteobacteria* | 41 | 124 |
|  |  | *Acidobacteriota* | 30 |  |
|  |  | *Chloroflexi* | 29 |  |
|  |  | *Actinobacteriota* | 11 |  |
|  |  | *Firmicutes* | 6 |  |
|  |  | *Gemmatimonadota* | 4 |  |
|  |  | *Dependentiae* | 1 |  |
|  |  | *Latescibacterota* | 1 |  |
|  |  | *Methylomirabilota* | 1 |  |
|  | June | *Proteobacteria* | 22 | 56 |
|  |  | *Acidobacteriota* | 15 |  |
|  |  | *Actinobacteriota* | 9 |  |
|  |  | *Chloroflexi* | 6 |  |
|  |  | *Firmicutes* | 2 |  |
|  |  | *Dependentiae* | 1 |  |
|  |  | *Gemmatimonadota* | 1 |  |
|  | Share | *Proteobacteria* | 23 | 64 |
|  |  | *Acidobacteriota* | 14 |  |
|  |  | *Chloroflexi* | 13 |  |
|  |  | *Actinobacteriota* | 6 |  |
|  |  | *Firmicutes* | 4 |  |
|  |  | *Gemmatimonadota* | 2 |  |
|  |  | *Myxococcota* | 2 |  |

Table S2 The affiliation of bacterial keystone taxa of three habitats in reservoir buffer strips from flooding to dry period.

| Type | Family | Phylum |
| --- | --- | --- |
| Cropland-April | *Solirubrobacteraceae* | *Actinobacteriota* |
|  | *Pyrinomonadaceae* | *Acidobacteriota* |
|  | *Rhizobiales_Incertae_Sedis* | *Proteobacteria* |
|  | *Intrasporangiaceae* | *Actinobacteriota* |
|  | *Hyphomicrobiaceae* | *Proteobacteria* |
|  | *Rhizobiaceae* | *Proteobacteria* |
| Cropland-June | *Vicinamibacteraceae* | *Acidobacteriota* |
|  | *Caulobacteraceae* | *Proteobacteria* |
|  | *f_unclassified_o__Subgroup_7* | *Acidobacteriota* |
|  | *f_unclassified_c__Alphaproteobacteria* | *Proteobacteria* |
|  | *Intrasporangiaceae* | *Actinobacteriota* |
|  | *Mycobacteriaceae* | *Actinobacteriota* |
|  | *Anaeromyxobacteraceae* | *Myxococcota* |
|  | *Hyphomicrobiaceae* | *Proteobacteria* |
|  | *f_unclassified_o__**Saccharimonadales* | *Patescibacteria* |
|  | *Xanthobacteraceae* | *Proteobacteria* |
| Grassland-April | *f_unclassified_c__Alphaproteobacteria* | *Proteobacteria* |
|  | *Acetobacteraceae* | *Proteobacteria* |
|  | *Xanthobacteraceae* | *Proteobacteria* |
|  | *Rhizobiales_Incertae_Sedis* | *Proteobacteria* |
|  | *Streptosporangiaceae* | *Actinobacteriota* |
|  | *f_unclassified_o__11−24* | *Acidobacteriota* |
|  | *Mycobacteriaceae* | *Actinobacteriota* |
|  | *Beijerinckiaceae* | *Proteobacteria* |
|  | *67−14* | *Actinobacteriota* |
|  | *Anaeromyxobacteraceae* | *Myxococcota* |
|  | *f_unclassified_o_Subgroup_17* | *Acidobacteriota* |
|  | *f_unclassified_o__Subgroup_7* | *Acidobacteriota* |
|  | *Roseiflexaceae* | *Chloroflexi* |
|  | *Reyranellaceae* | *Proteobacteria* |
| Grassland-June | *Microscillaceae* | *Bacteroidota* |
|  | *Bacillaceae* | *Firmicutes* |
|  | *Blastocatellaceae* | *Acidobacteriota* |
|  | *Diplorickettsiaceae* | *Proteobacteria* |
|  | *f_unclassified_o__Vicinamibacterales* | *Acidobacteriota* |
|  | *Nitrosomonadaceae* | *Proteobacteria* |
|  | *f_unclassified_o__IMCC26256* | *Actinobacteriota* |
|  | *f_unclassified_o__C0119* | *Chloroflexi* |
|  | *f_unclassified_o__Rokubacteriales* | *Methylomirabilota* |
|  | *f_unclassified_o__Subgroup_17* | *Acidobacteriota* |
|  | *Nitrospiraceae* | *Nitrospirota* |
| Woodland-April | *f_unclassified_o__Microtrichales* | *Actinobacteriota* |
|  | *f_unclassified_c__MB−A2−108* | *Actinobacteriota* |
|  | *f_unclassified_o__**S085* | *Chloroflexi* |
|  | *Hyphomicrobiaceae* | *Proteobacteria* |
|  | *Clostridiaceae* | *Firmicutes* |
|  | *Pyrinomonadaceae* | *Acidobacteriota* |
|  | *Pseudonocardiaceae* | *Actinobacteriota* |
| Woodland-June | *f_unclassified_o__Vicinamibacterales* | *Acidobacteriota* |
|  | *Intrasporangiaceae* | *Actinobacteriota* |
|  | *Reyranellaceae* | *Proteobacteria* |
|  | *Myxococcaceae* | *Myxococcota* |
|  | *Chthoniobacteraceae* | *Verrucomicrobiota* |
|  | *Solirubrobacteraceae* | *Actinobacteriota* |
|  | *f_unclassified_c__**KD4−96* | *Chloroflexi* |
|  | *f_unclassified_o__SBR1031* | *Chloroflexi* |
|  | *Gaiellaceae* | *Actinobacteriota* |
|  | *f_unclassified_o__S085* | *Chloroflexi* |
|  | *f_unclassified_c__bacteriap25* | *Myxococcota* |
